# Supplementary material for: Concentration-induced spontaneous polymerization of protic ionic liquids for efficient in situ adhesion
Source: Nat Commun. 2024 May 20;15:4265. doi: 10.1038/s41467-024-48561-1 (PMC11106314; doi:10.1038/s41467-024-48561-1)
Supplement: Supplementary file 1 — Supplementary Information [file 41467_2024_48561_MOESM1_ESM.pdf]

Supplementary Information

Concentration-induced spontaneous polymerization of protic ionic liquids for efficient in situ adhesion

Jun Zhang[1], Xuan Zhou[1], Qinyu Hu[1], Kaijian Zhou[1], Yan Zhang[1], Shengyi Dong[2], Gai Zhao[3], and Shiguo Zhang[1]*

[1]College of Materials Science and Engineering, Hunan University, Changsha 410004, China

[2]College of Chemistry and Chemical Engineering, Hunan University, Changsha 410082, China

[3]State Key Laboratory of Mechanics and Control of Aerospace Structures, Nanjing University of Aeronautics and Astronautics, Nanjing 210016, China

*Corresponding author. Email: zhangsg@hnu.edu.cn

Supplementary Methods

Preparation of MXene

0.3 g of 1,4-Naphthalenedicarboxylic acid was dissolved in 30 mL of deionized water. Then, 0.08 g of Ti_3AlC_2 powder and 1.5 mL of 40 wt% HF were added to the above solution to form a homogeneous suspension under magnetic stirring. The suspension was transferred into a 50 ml Teflon-lined autoclave and heated at 160 °C for 6 h. The obtained sediment was washed with N, N-Dimethylformamide, and water till the pH was neutral, and dried under vacuum at 60 ℃ for 24 h. After this period, the MXene was obtained (yield: 47%).

Preparation of rGO

2 g of graphite was added into 10 mL of concentrated H_2SO_4, then 1.7 g of $K_2S_2O_8$ and 1.7 g of P_2O_5. The mixture was heated to 80 ℃ and stirred for 5 h. It was then diluted with 300 mL of deionized water. The product was filtered, washed with water 5 times, and dried. Put about 1 g of obtained pre-oxidized graphite into 35 mL of concentrated H_2SO_4 with mechanical stirring. 5 g of $KMnO_4$ powder was slowly added under an ice bath. After being stirred at 35 ℃ for 8 h, the product was transferred into 250 mL of DIW, and then 3 mL of 30% H_2O_2 was added dropwise. The mixture was filtered and washed with a 1:10 aqueous solution of HCl (250 mL) to remove metal ions. The product was collected by centrifugation, washed with water 5 times, and freezing-dried under a vacuum to obtain the GO (yield: 70%). Subsequently, the GO was reduced under 1000 ℃ for 2 h in an Ar atmosphere with the heating rate of 10 ℃ min^{-1}. After this period, the rGO was obtained (yield: 90%).

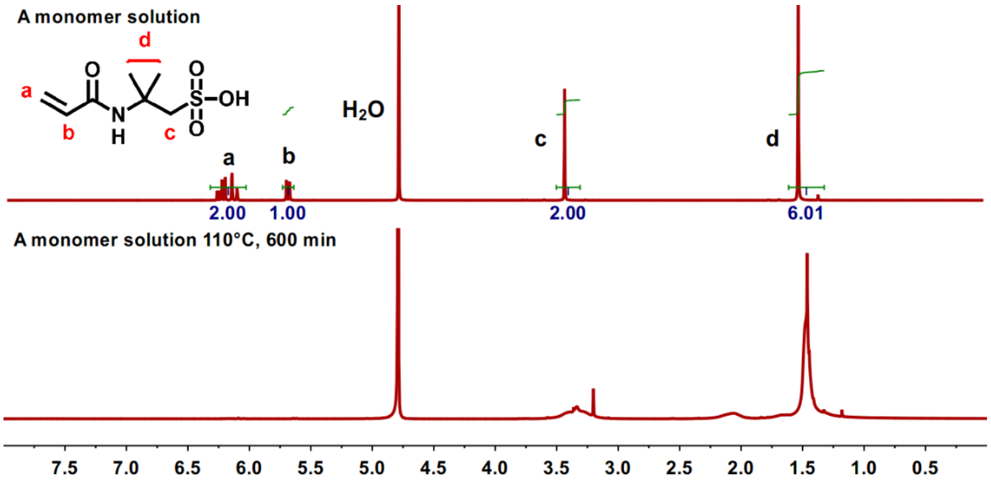

Supplementary Fig. 1. ^{1}H NMR spectrum (400 MHz, 25 ℃, D_2O) of a monomer solution with a concentration of 1 M before and after isothermal curing treatment (110 ℃, 600 min).

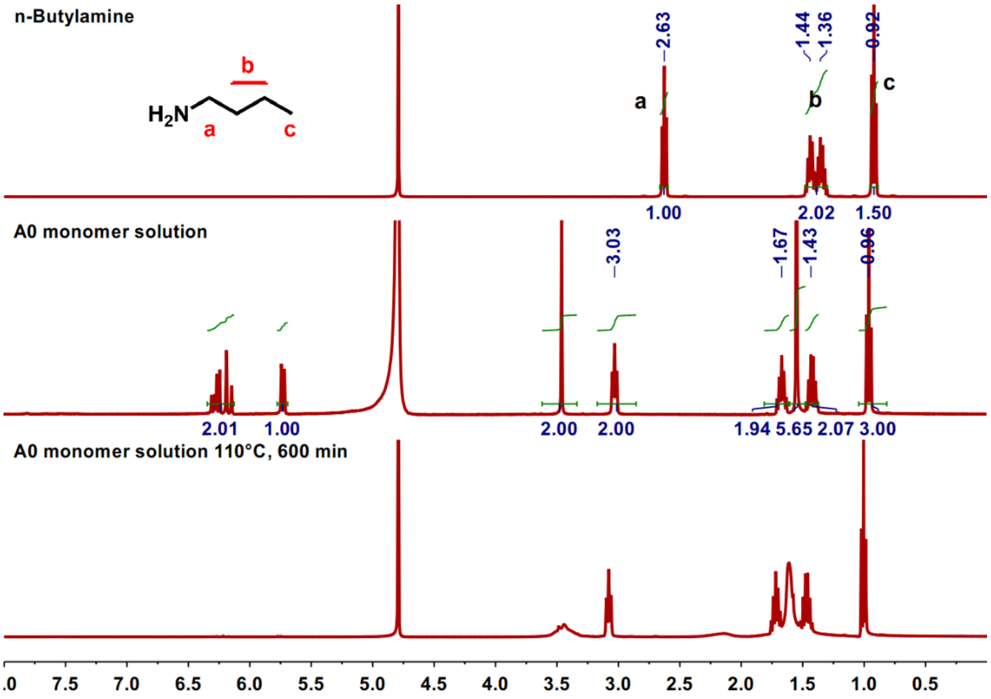

Supplementary Fig. 2. ^{1}H NMR spectrum (400 MHz, 25 ℃, D_2O) of A0 monomer solution with a concentration of 1 M before and after isothermal curing treatment (110 ℃, 600 min).

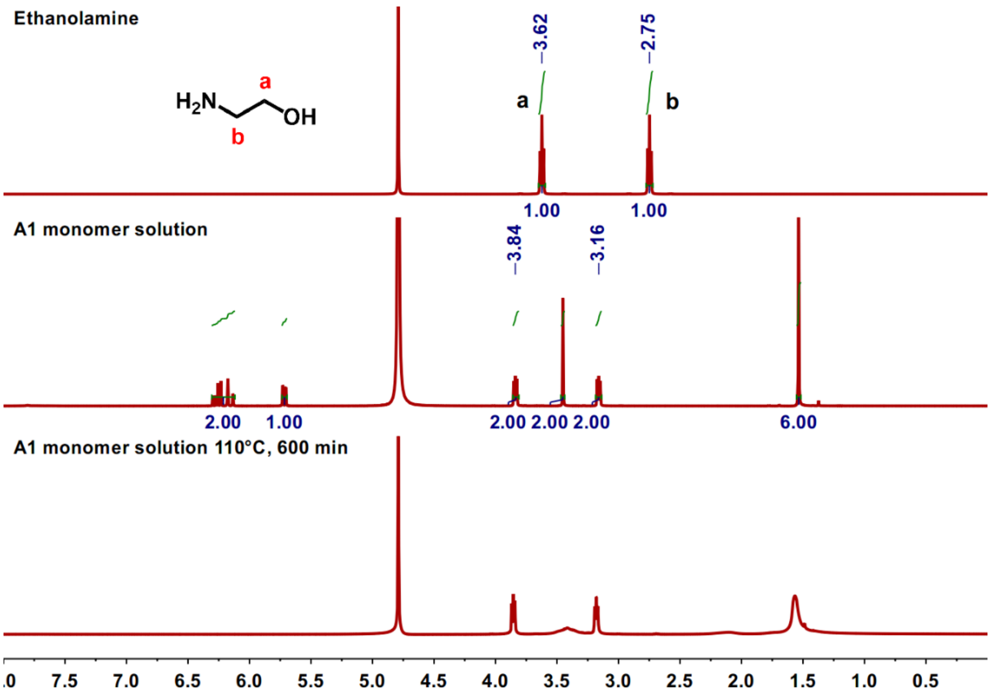

Supplementary Fig. 3. ^{1}H NMR spectrum (400 MHz, 25 ℃, D_2O) of A1 monomer solution with a concentration of 1 M before and after isothermal curing treatment (110 ℃, 600 min).

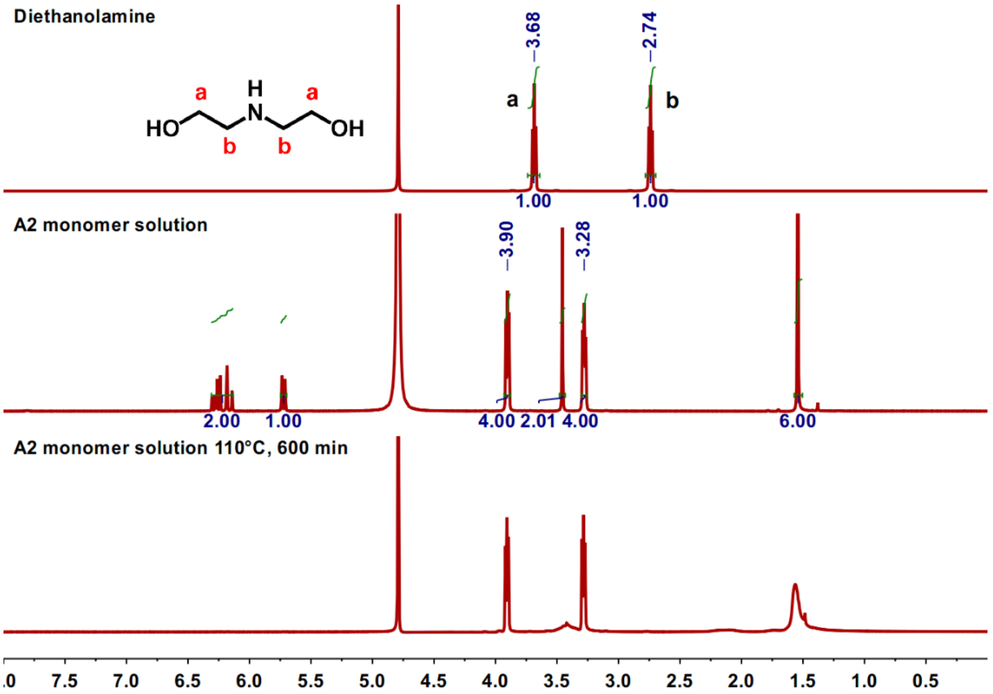

Supplementary Fig. 4. ^{1}H NMR spectrum (400 MHz, 25 ℃, D_2O) of A2 monomer solution with a concentration of 1 M before and after isothermal curing treatment (110 ℃, 600 min).

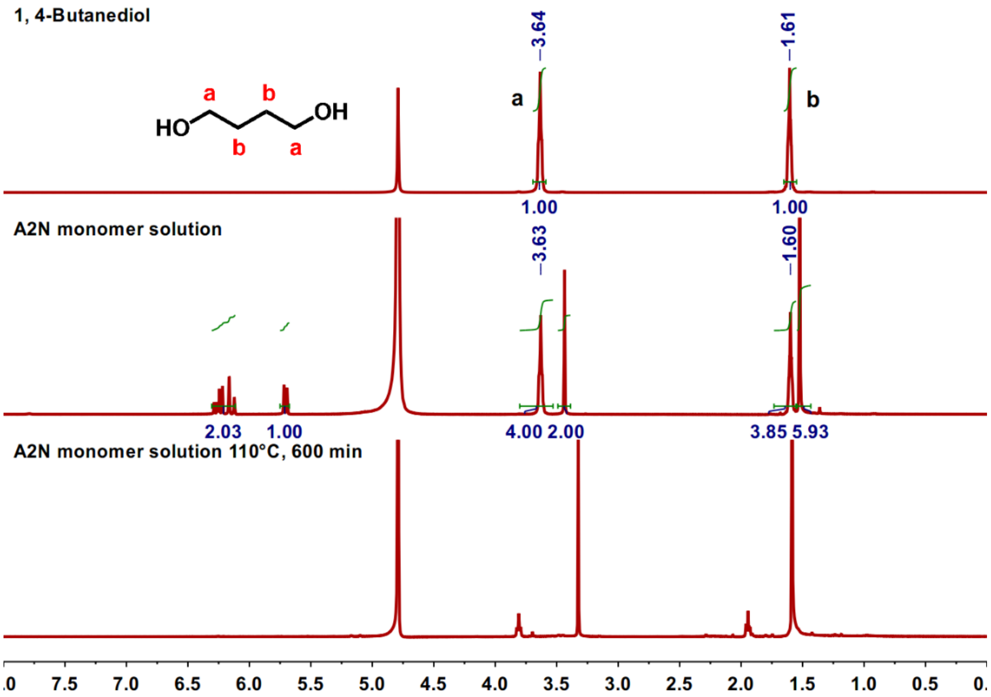

Supplementary Fig. 5. ^{1}H NMR spectrum (400 MHz, 25 ℃, D_2O) of A2N monomer solution with a concentration of 1 M before and after isothermal curing treatment (110 ℃, 600 min).

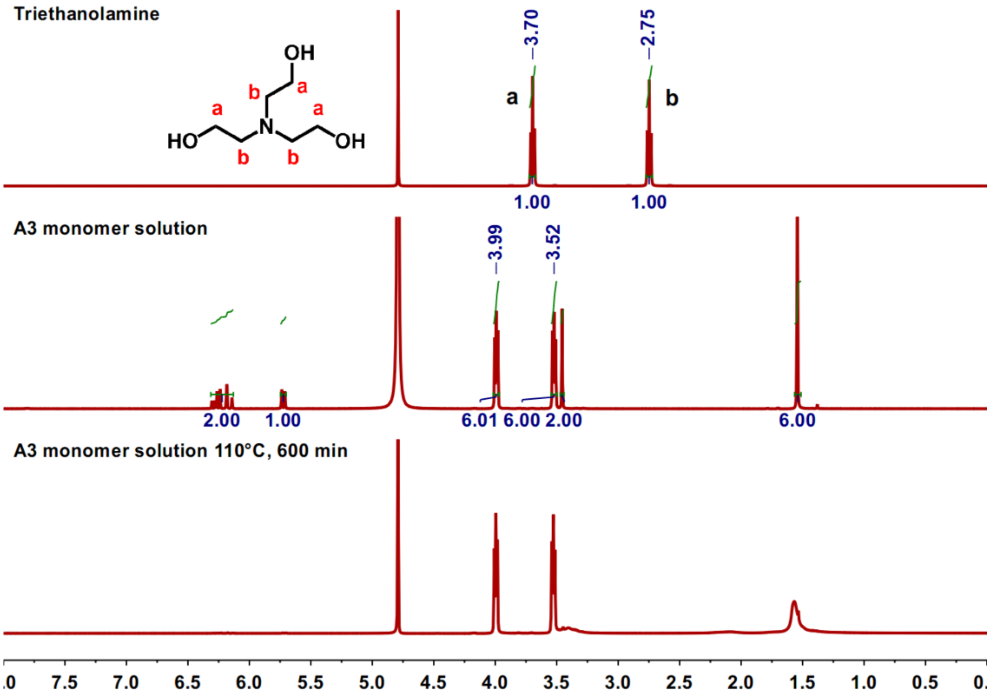

Supplementary Fig. 6. ^{1}H NMR spectrum (400 MHz, 25 ℃, D_2O) of A3 monomer solution with a concentration of 1 M before and after isothermal curing treatment (110 ℃, 600 min).

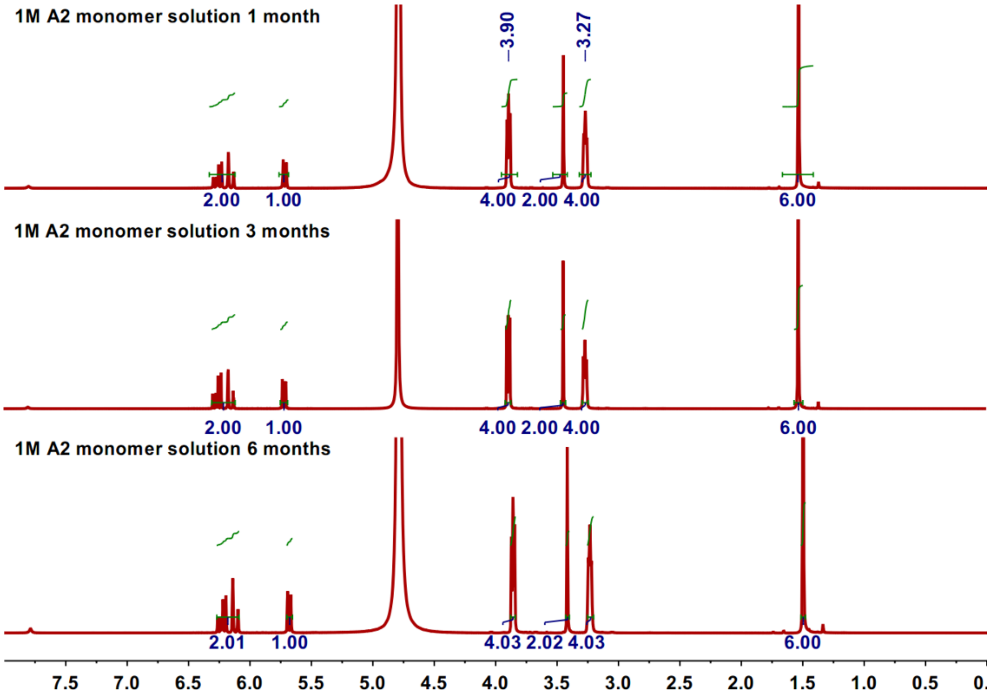

Supplementary Fig. 7. ^1H NMR spectrum (400 MHz, 25 ℃, D_2O) of A2ms with a concentration of 1 M after being preserved for different months at 25 ℃.

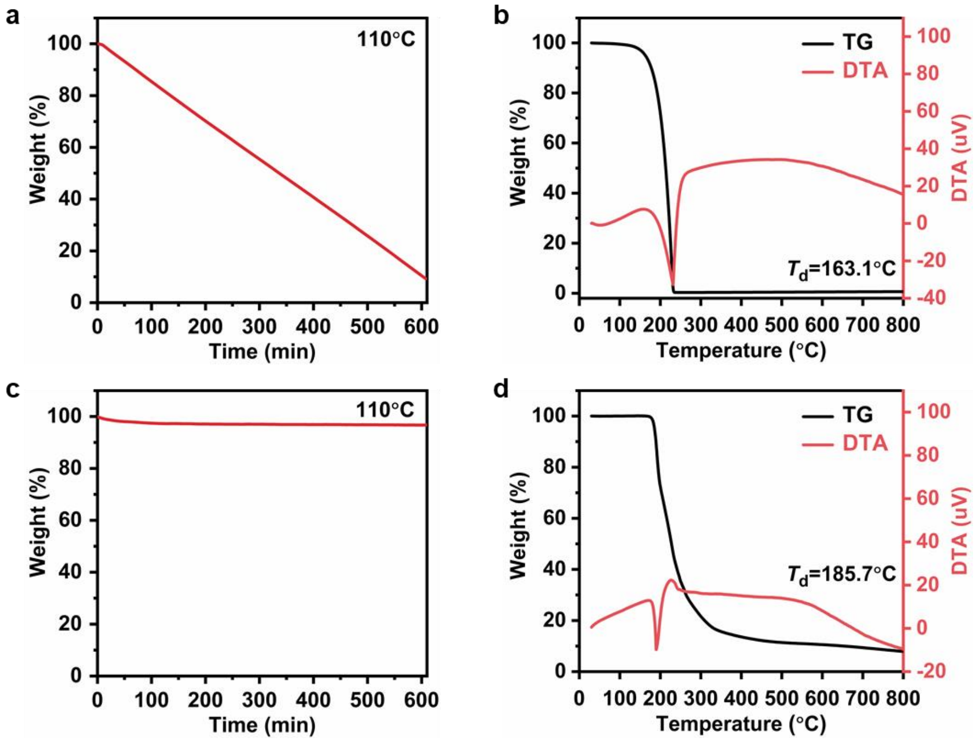

Supplementary Fig. 8. Isothermal TGA and TGA curves. (a) and (b) DEA, (c) and (d) AMPS (T_d is defined as the decomposition temperature when 5% weight loss).

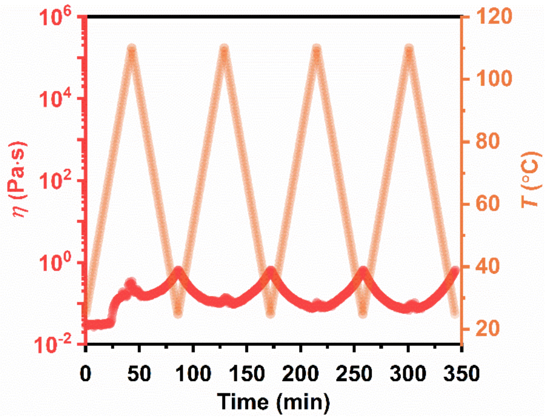

Supplementary Fig. 9. Reversible temperature-dependent rheological test of the reference solution without AMPS monomers (i.e., 1 M DEA solution, angular frequency: 10 rad s^{-1}, strain: 1%). In the following cyclic rheological test, the viscosity of the reference solution was reversible, indicating the absence of any reaction.

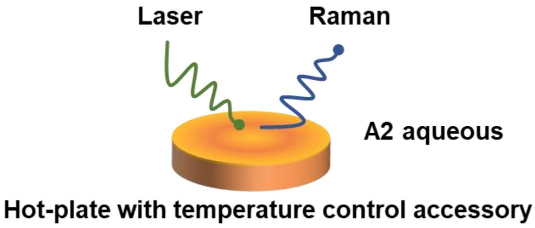

Supplementary Fig. 10. Schematic of in-situ temperature-dependent Raman characterization (A2ms was evenly cast on the hot plate).

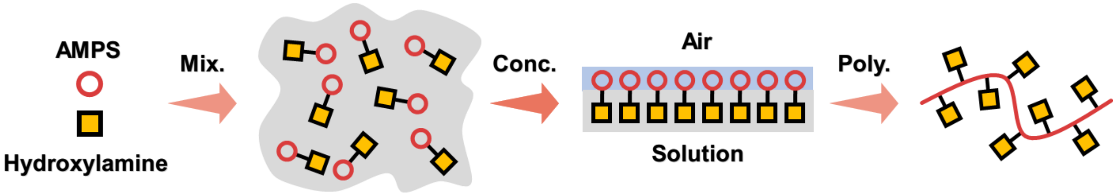

Supplementary Fig. 11. Schematic of the spontaneous polymerization in the concentration process.

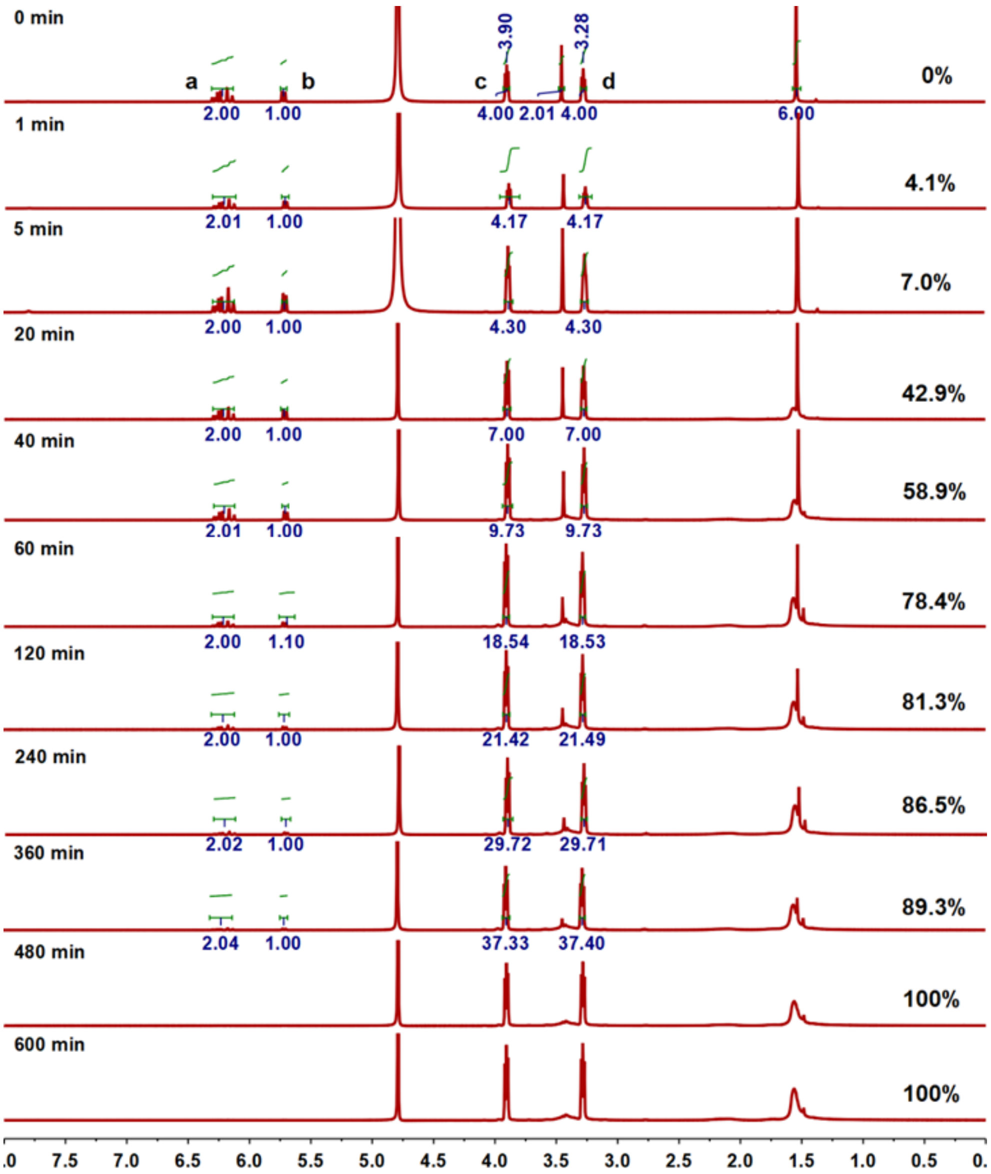

Supplementary Fig. 12. ^{1}H NMR spectrum (400 MHz, 25 ℃, D_2O) of A2ms after isothermal curing treatment for different times at 110 ℃. The conversion rate of monomers of A2 and other reference samples can be calculated using the following equation: $(1 – 4 * S_b / S_c) * 100\%$, wherein S_a and S_b are the integral areas of the vinyl groups, S_c and S_d are the integral areas of CH_2 groups of diethanolamine molecules. Relationships of $S_a \approx 2 * S_b$ and $S_c \approx S_d$ were controlled to ensure integral accuracy.

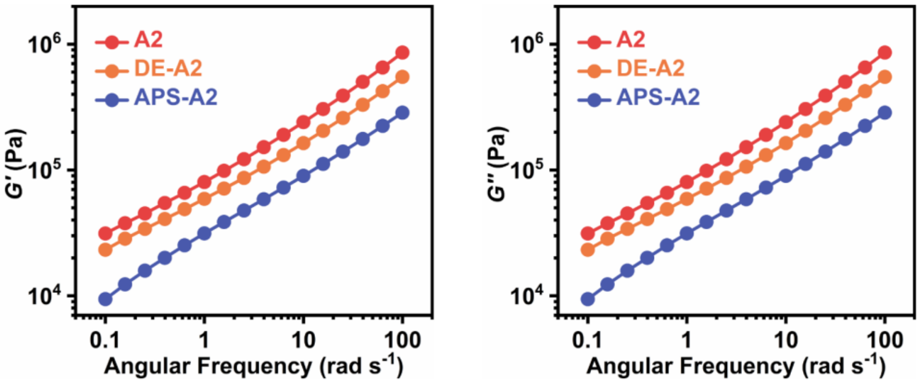

Supplementary Fig. 13. Frequency-dependent rheological test of A2 and reference samples with partial disentanglement (DE-A2) or polymerized using initiator of ammonium persulfate (APS-A2).

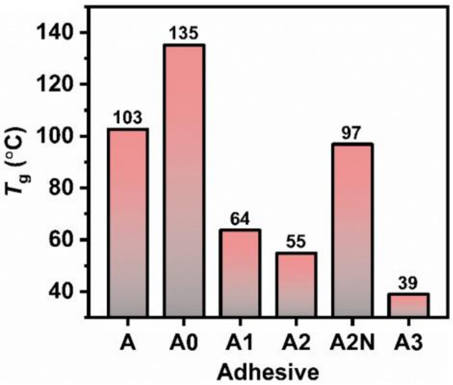

Supplementary Fig. 14. The glass transition temperature of A2 and reference samples.

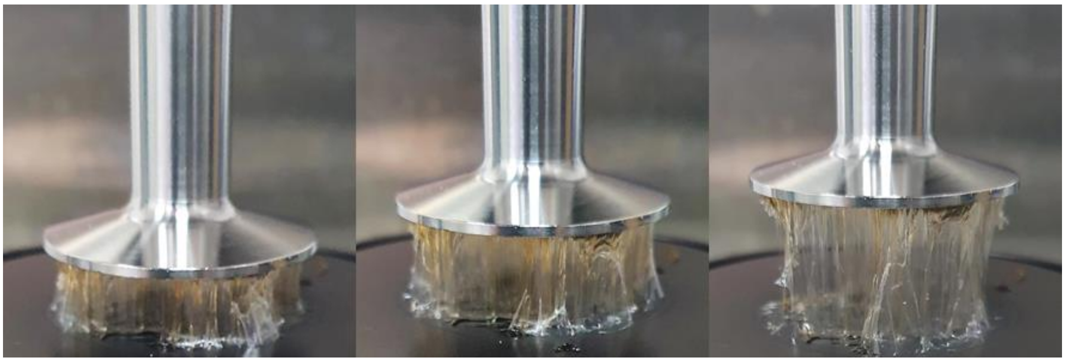

Supplementary Fig. 15. Photograph of the wire-drawing process of A2 at 110 ℃.

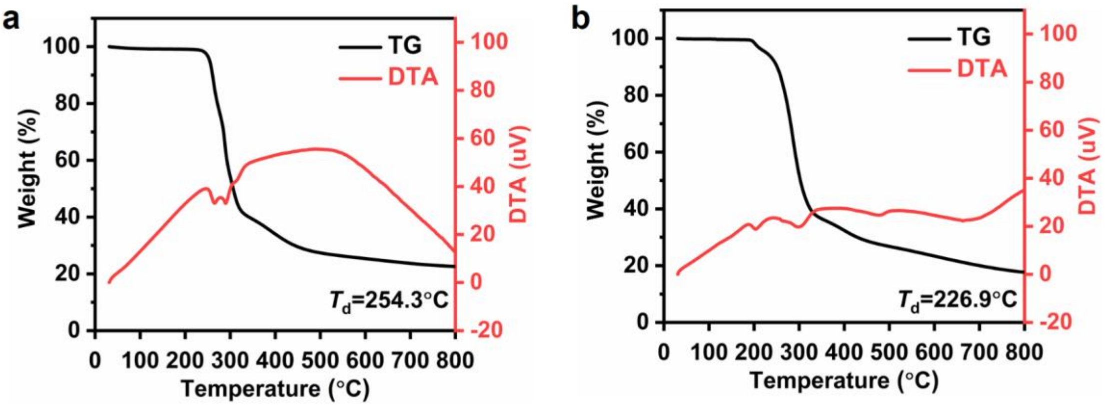

Supplementary Fig. 16. TGA curves. Spontaneously polymerized (a) A2 and (b) A (T_d is defined as the decomposition temperature when 5% weight loss).

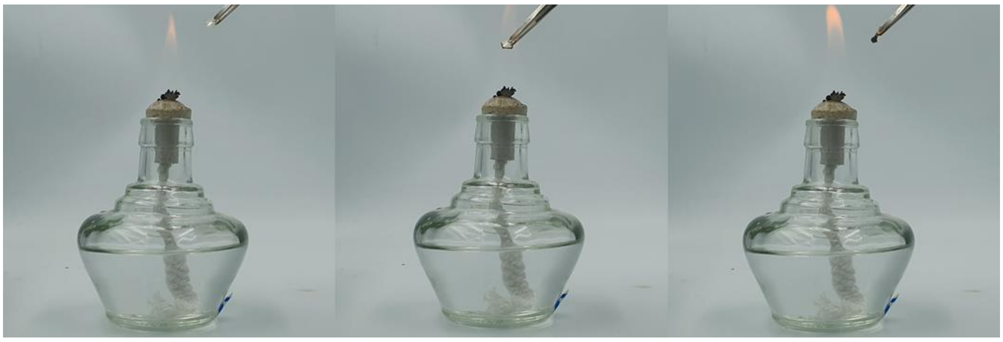

Supplementary Fig. 17. Photographs of A2 ignited by flames.

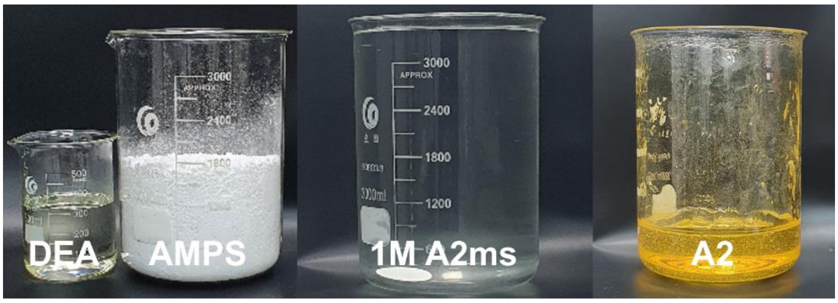

Supplementary Fig. 18. Large scale preparation of A2 (weight of obtained A2: 1 kg).

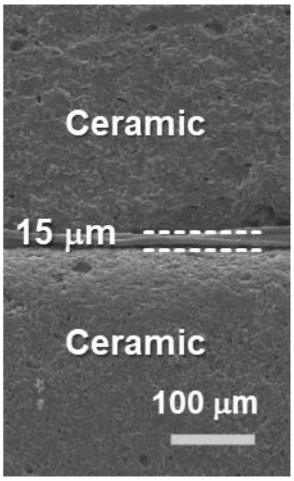

Supplementary Fig. 19. The thickness of two adhered ceramic substrates using in-situ spontaneous polymerization of A2ms.

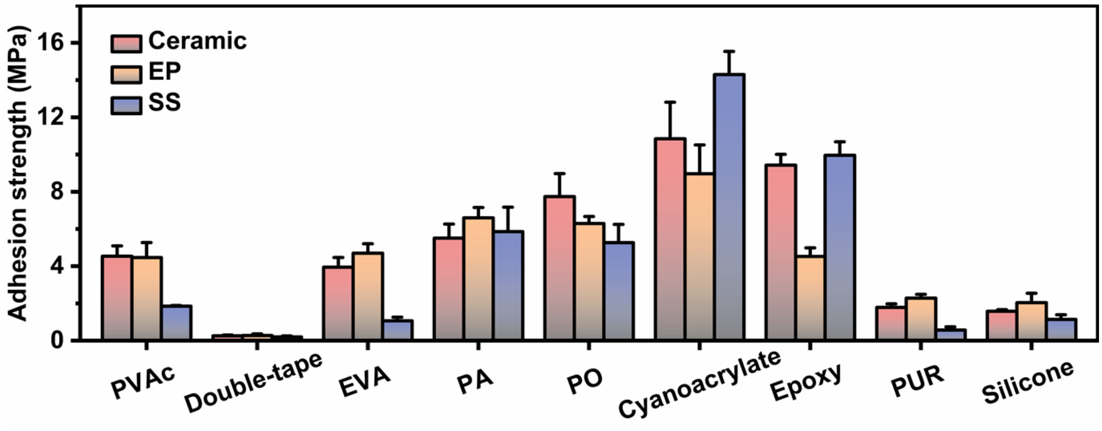

Supplementary Fig. 20. Adhesion strengths of commercially available adhesives. All data are presented as mean ± SD (n = 3~5 independent samples).

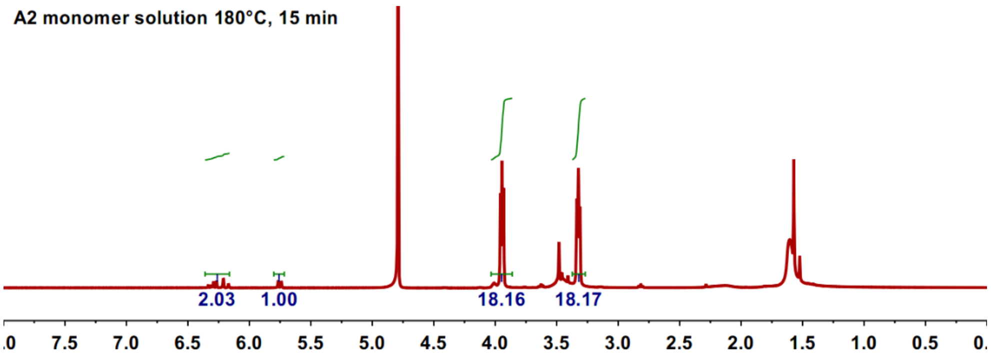

Supplementary Fig. 21. ^{1}H NMR spectrum (400 MHz, 25 ℃, D_2O) of A2 monomer solution with a concentration of 1 M after isothermal curing treatment for 15 min at 180 ℃.

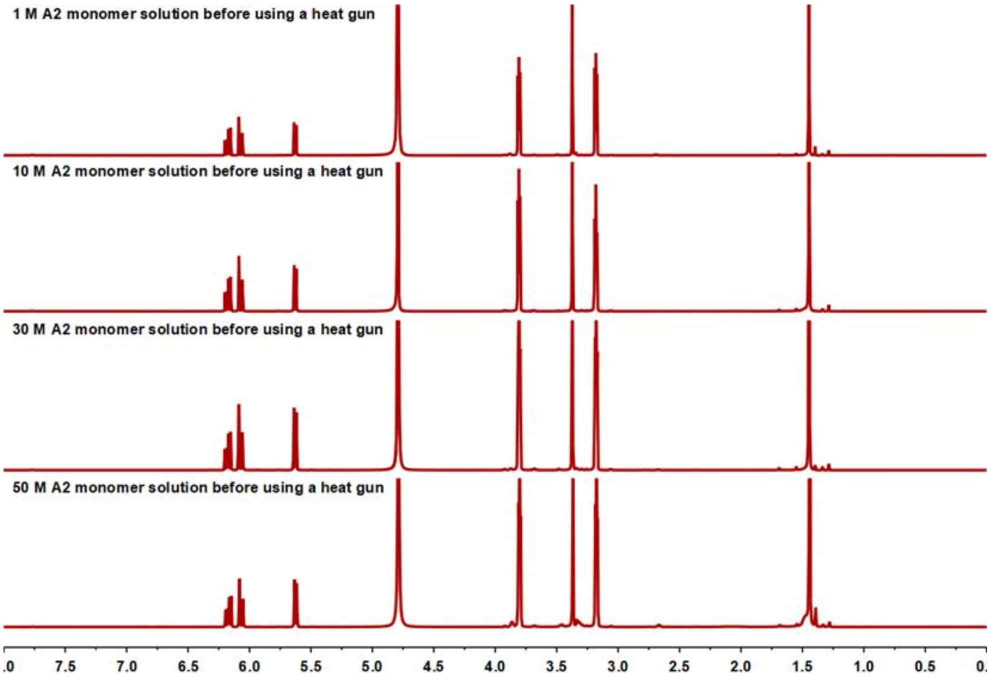

Supplementary Fig. 22. ^{1}H NMR spectrum (400 MHz, 25 ℃, D_2O) of A2 monomer solution with different initial concentration before using a higher-temperature laboratory heat gun.

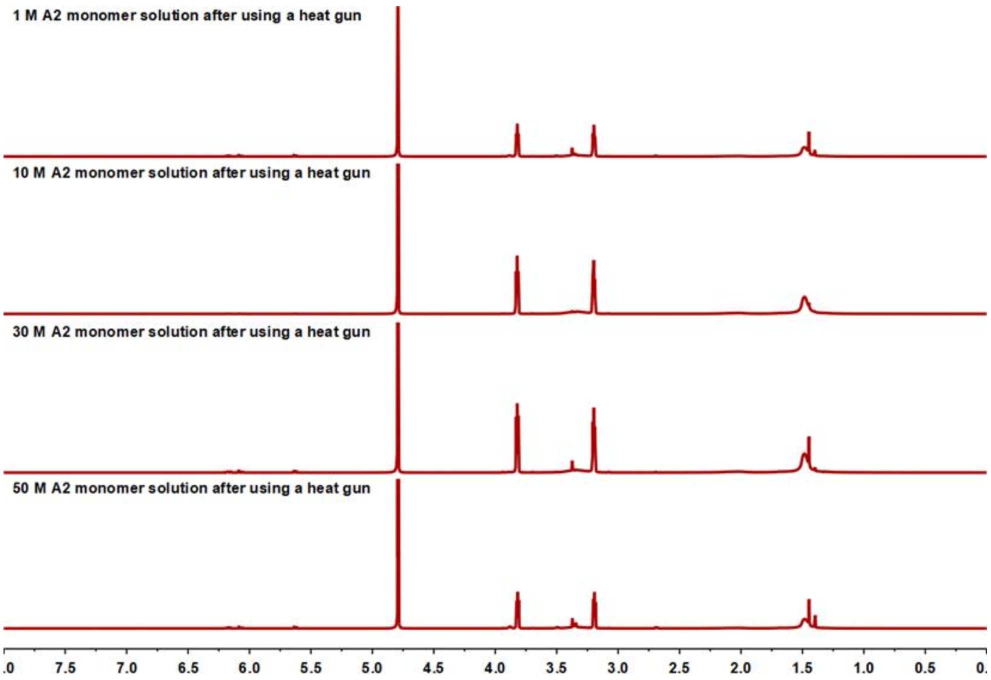

Supplementary Fig. 23. ^{1}H NMR spectrum (400 MHz, 25 ℃, D_2O) of A2 monomer solution with different initial concentration after using a higher-temperature laboratory heat gun.

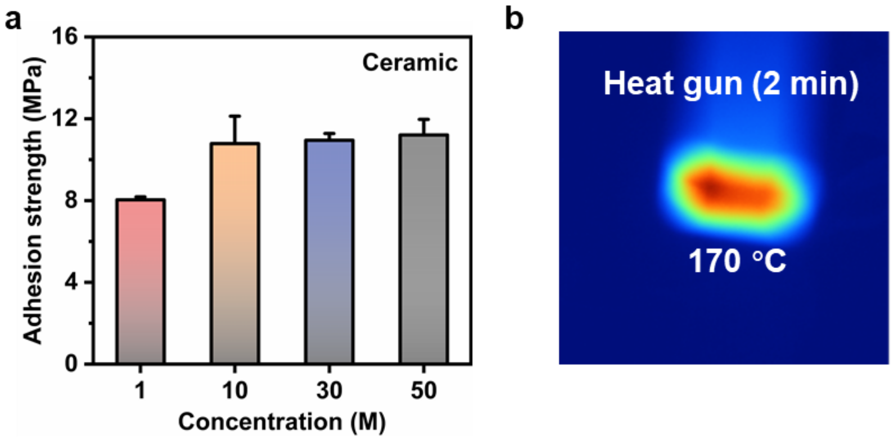

Supplementary Fig. 24. In-situ polymerization of A2 using a higher-temperature laboratory heat gun. (a) Adhesion strengths of A2 with different initial monomer concentrations obtained by polymerization conditions using a higher-temperature laboratory heat gun (roughly 170 °C) for 2 min. All data are presented as mean ± SD (n = 3~5 independent samples). (b) The temperature detected by IR thermal imager for ceramic substrate with A2 under laboratory heat gun for 2 min.

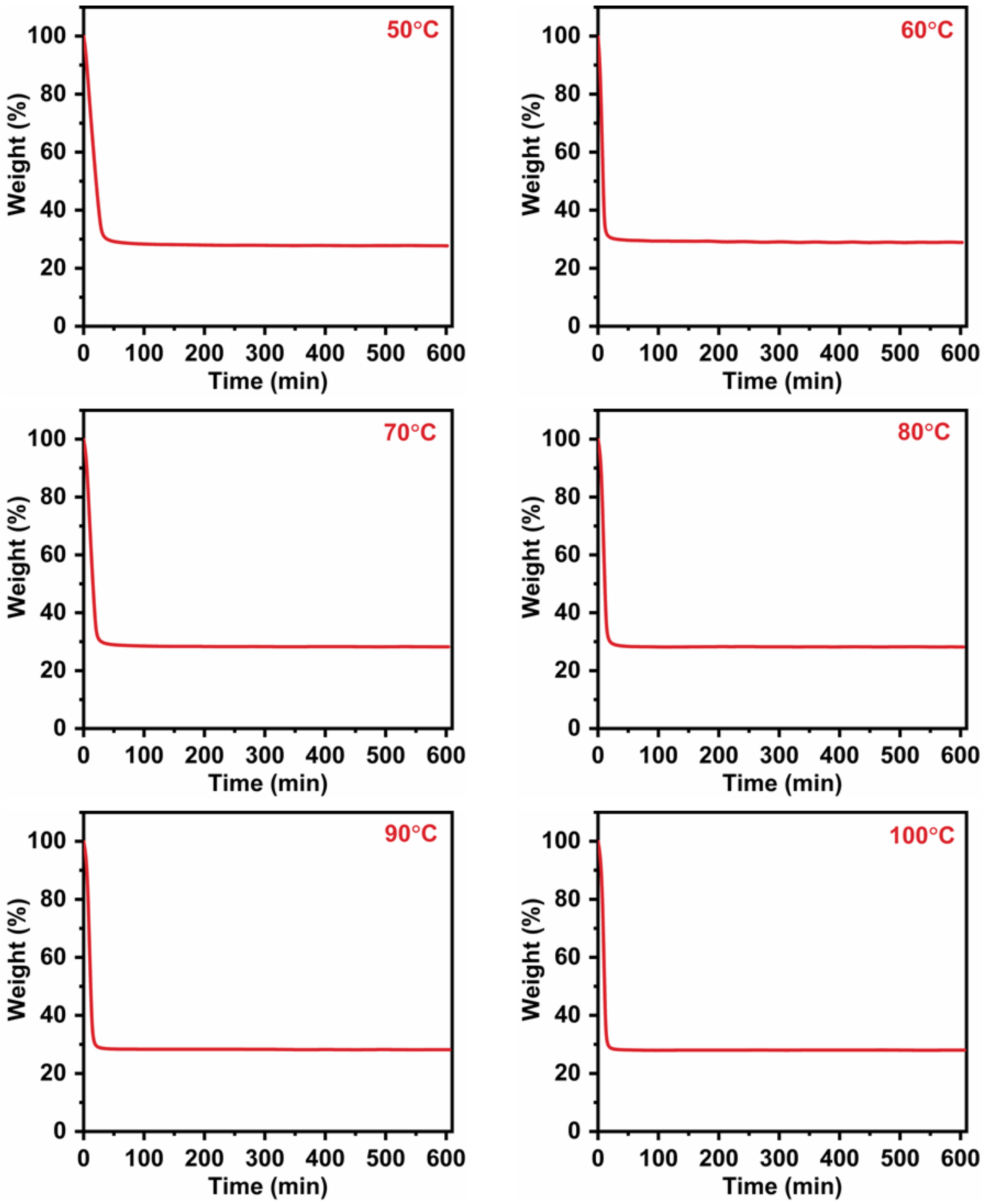

Supplementary Fig. 25. Isothermal TGA of A2 monomer solution (concentration: 1 M) at different temperatures.

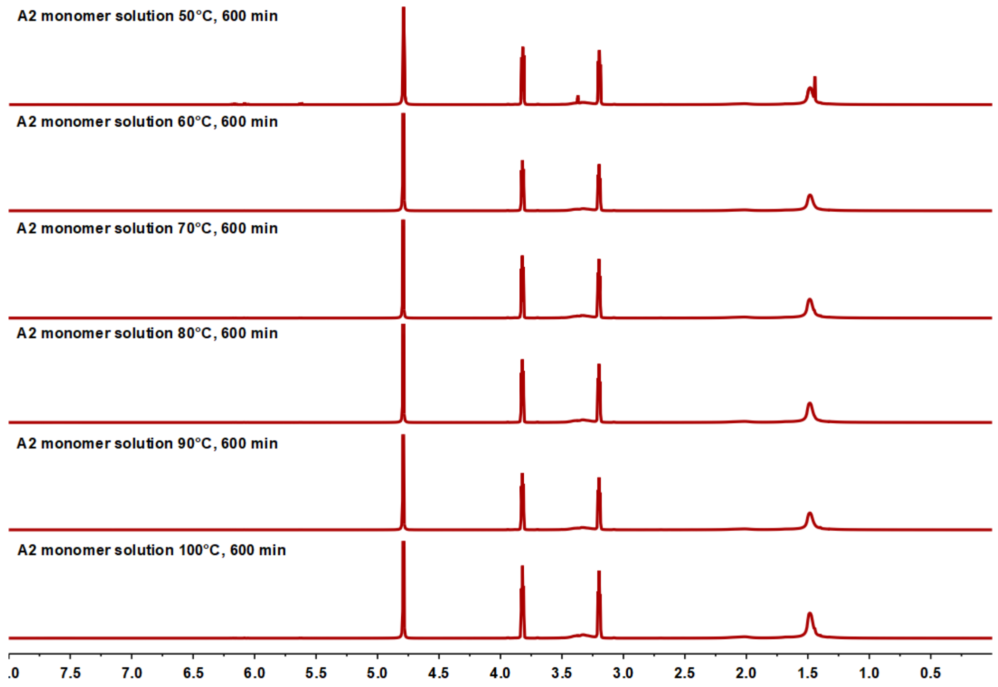

Supplementary Fig. 26. ^{1}H NMR spectrum (400 MHz, 25 ℃, D_2O) of A2 monomer solution with a concentration of 1 M after different temperatures in isothermal curing treatments.

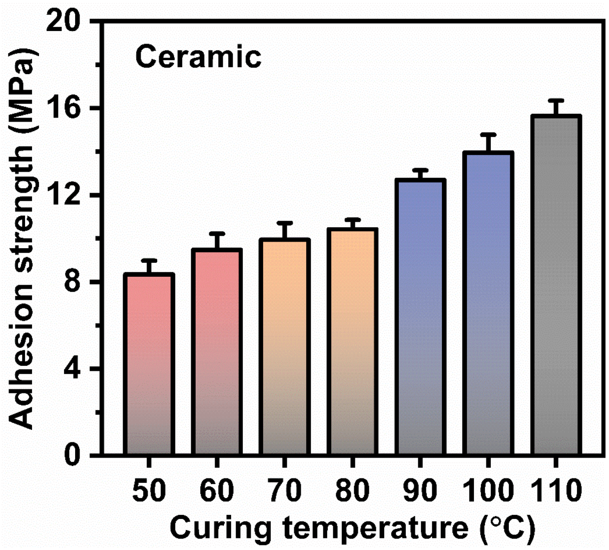

Supplementary Fig. 27. Adhesion strengths of A2 obtained by spontaneous polymerization with different temperatures in isothermal curing treatments. All data are presented as mean ± SD (n = 3~5 independent samples).

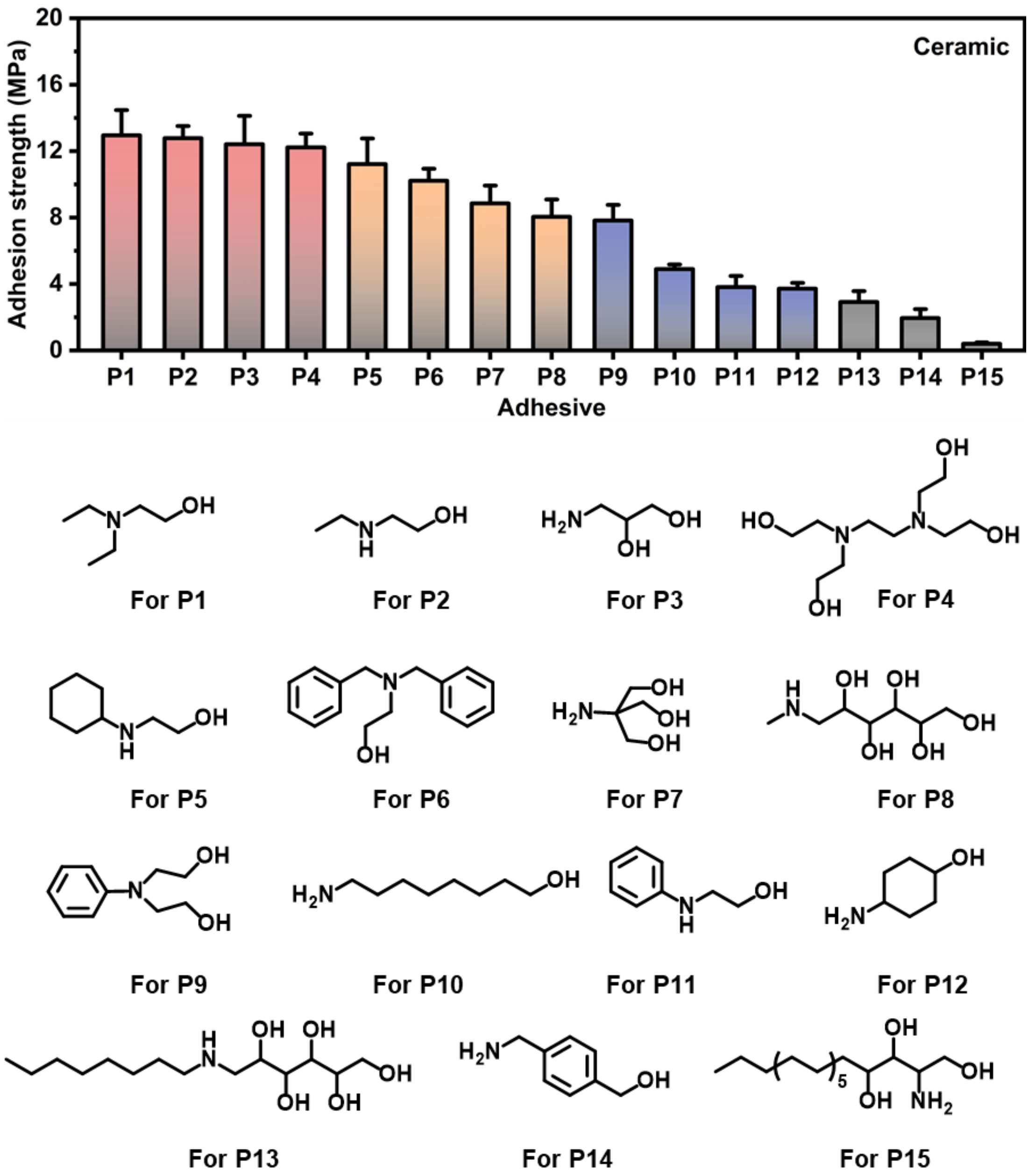

Supplementary Fig. 28. Adhesion strengths of PPILs obtained by different hydroxylamine molecules. All data are presented as mean ± SD (n = 3~5 independent samples).

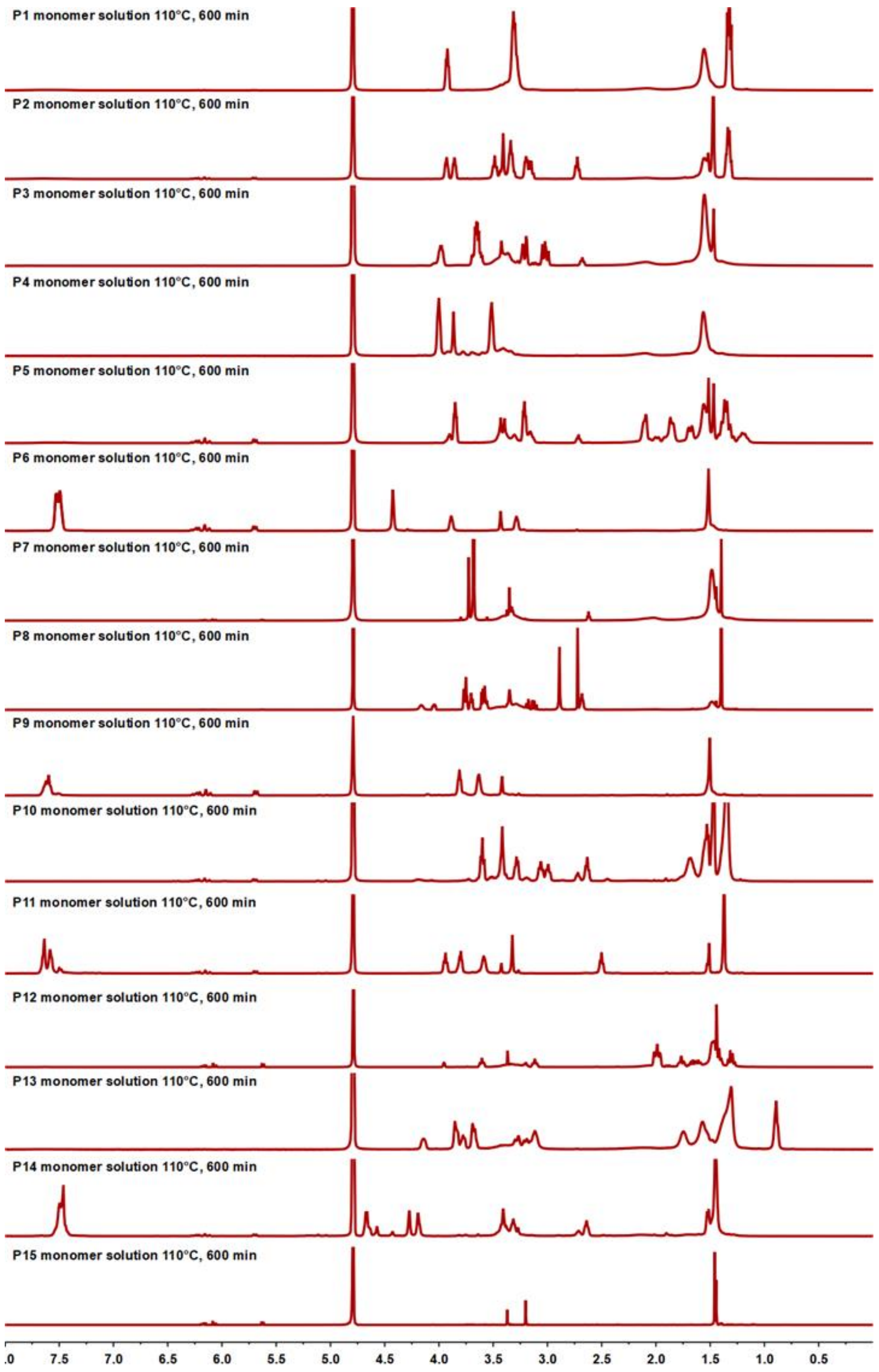

Supplementary Fig. 29. ^{1}H NMR spectrum (400 MHz, 25 ℃, D_2O) of PPIL obtained by different hydroxylamine after isothermal curing treatment for different times at 110 ℃.

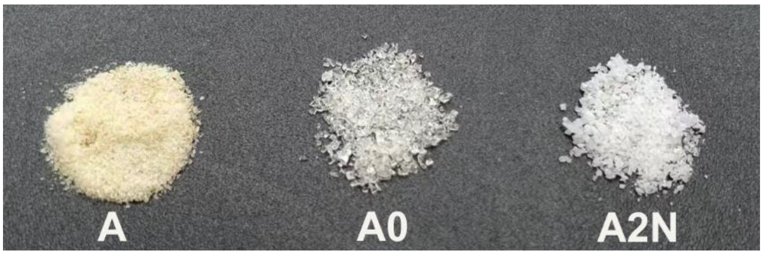

Supplementary Fig. 30. Photographs of reference samples of spontaneously polymerized A, A0, and A2N under ambient conditions (temperature: 25 ℃).

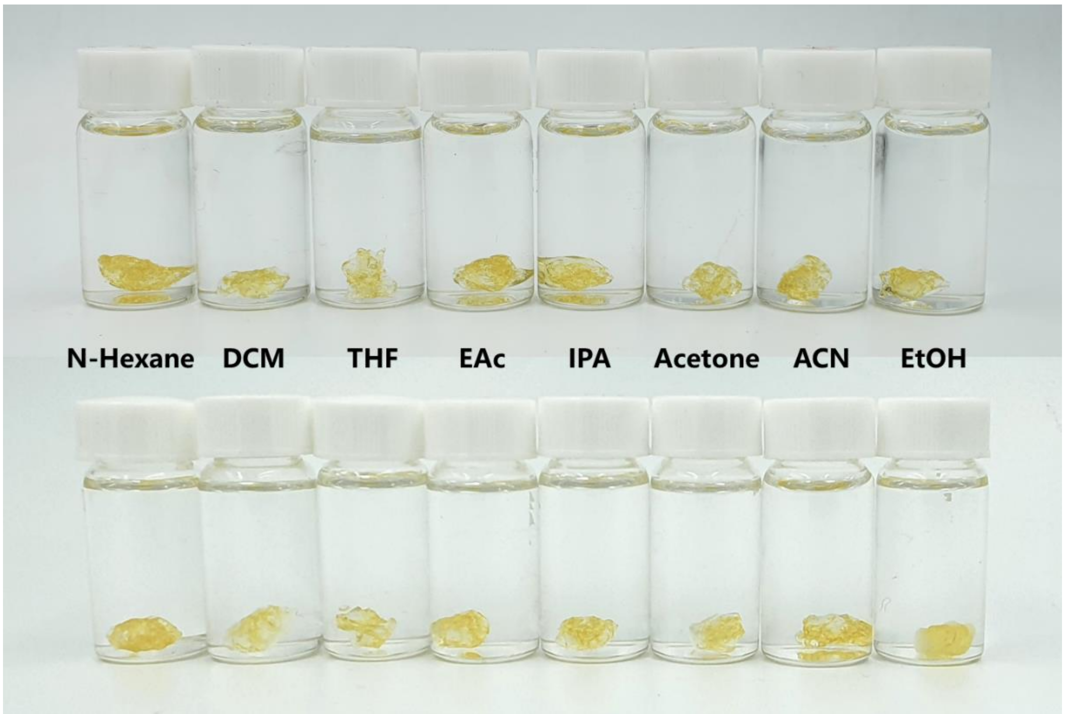

Supplementary Fig. 31. The solubility test of A2 before (top) and after (bottom) soaking for 1 month in various organic solvents.

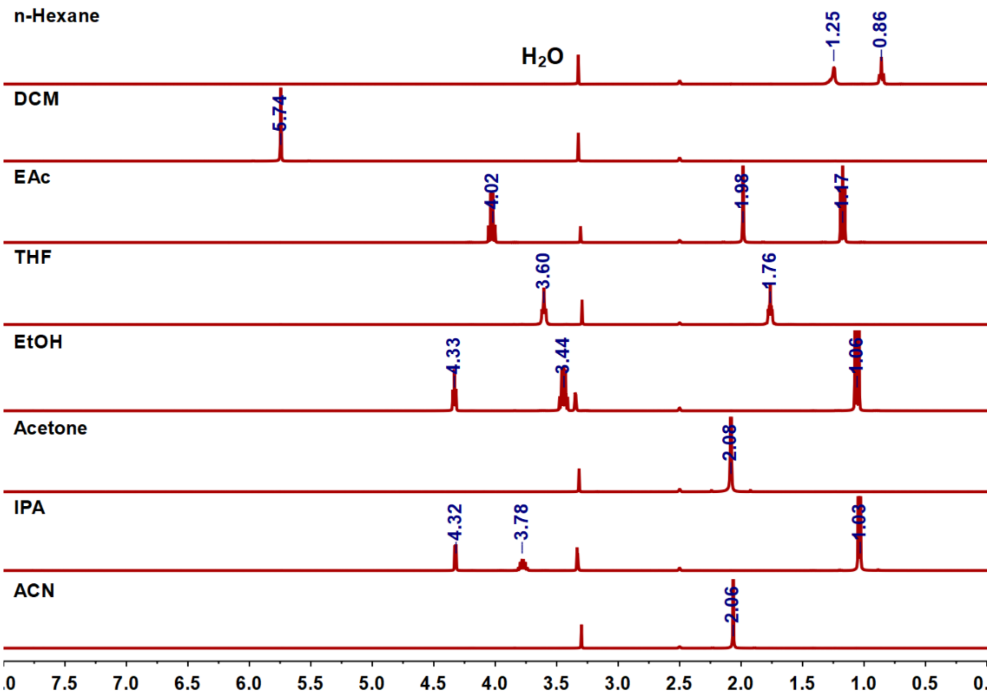

Supplementary Fig. 32. ^{1}H NMR spectrum (400 MHz, 25 ℃, DMSO-d_6) of various organic solvents after A2 being immersed for 1 month in Supplementary Fig. 31.

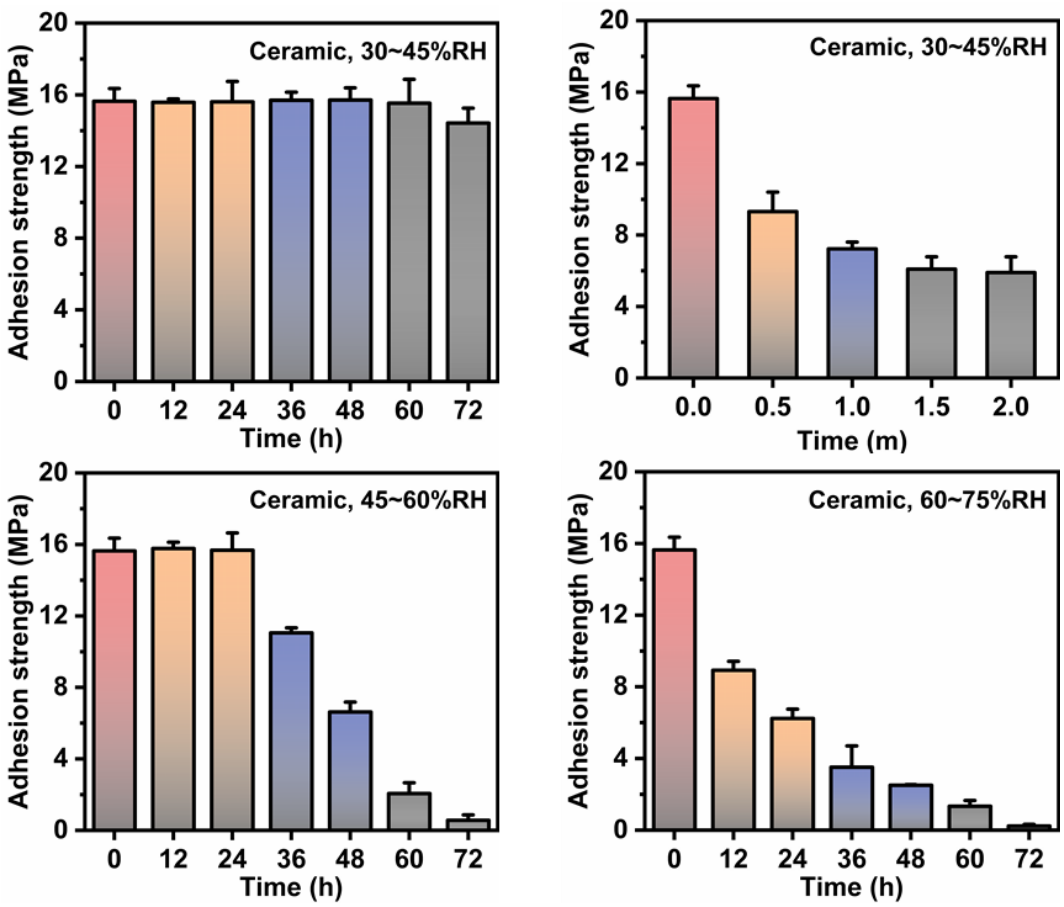

Supplementary Fig. 33. Adhesion strengths of A2 after storing in environmental conditions with different humidity. All data are presented as mean ± SD (n = 3~5 independent samples).

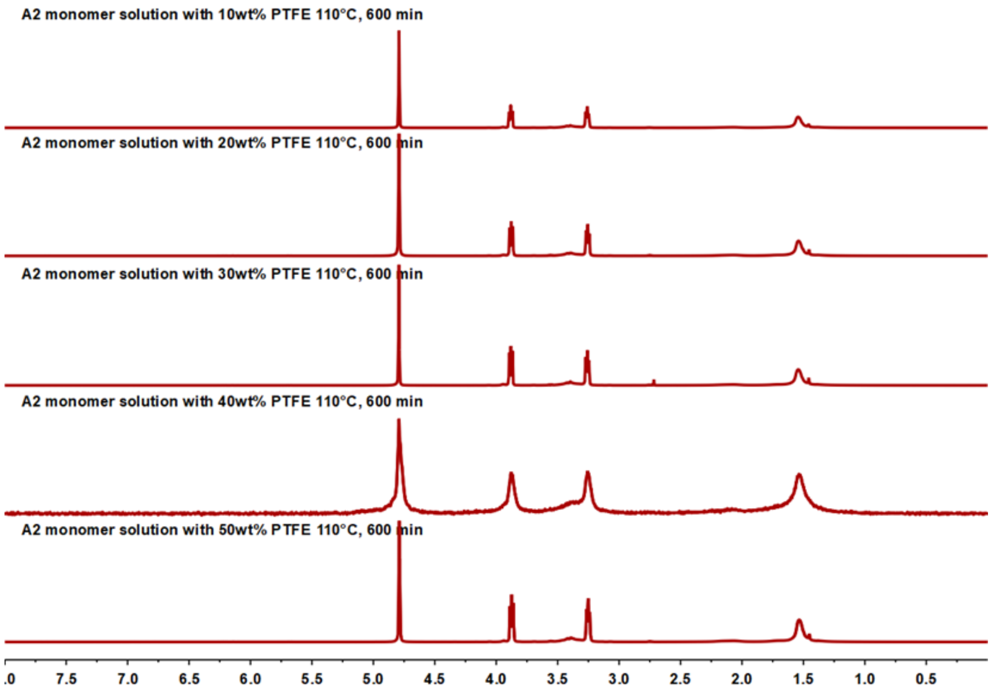

Supplementary Fig. 34. ^{1}H NMR spectrum (400 MHz, 25 ℃, D_2O) of A2 monomer solution with different amount of PTFE before isothermal curing treatment (110 ℃, 600 min).

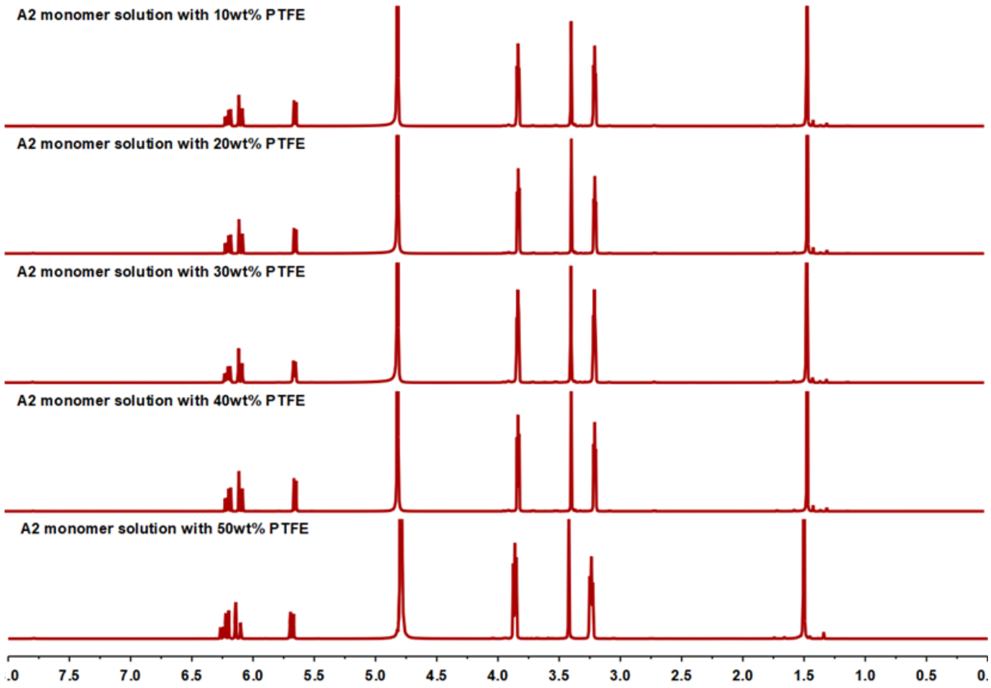

Supplementary Fig. 35. ^{1}H NMR spectrum (400 MHz, 25 ℃, D_2O) of A2 monomer solution with different amount of PTFE after isothermal curing treatment (110 ℃, 600 min).

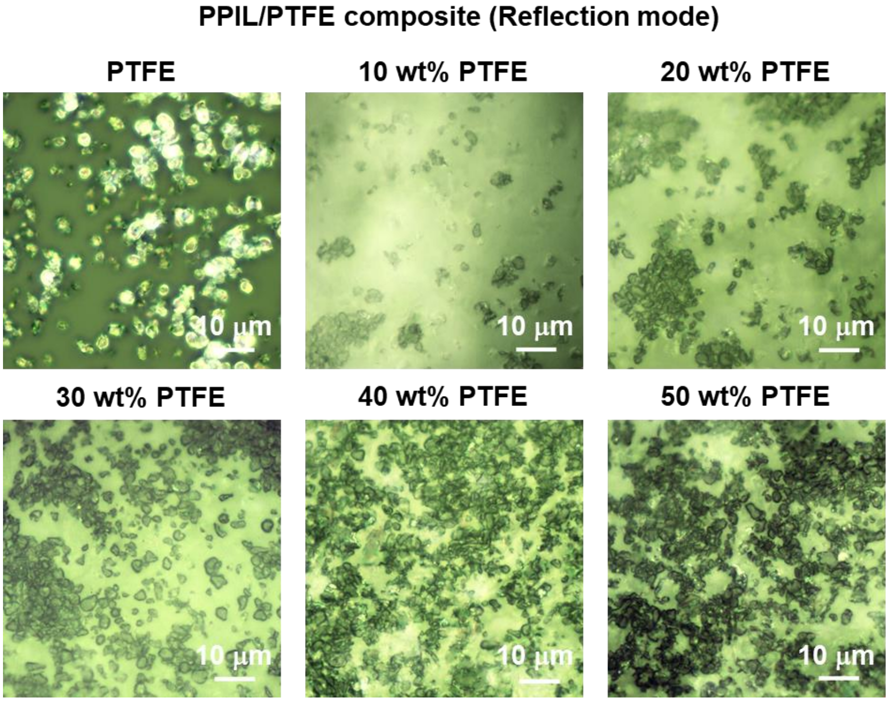

Supplementary Fig. 36. Light microscope (reflection mode) images of the PTFE and PPIL/PTFE.

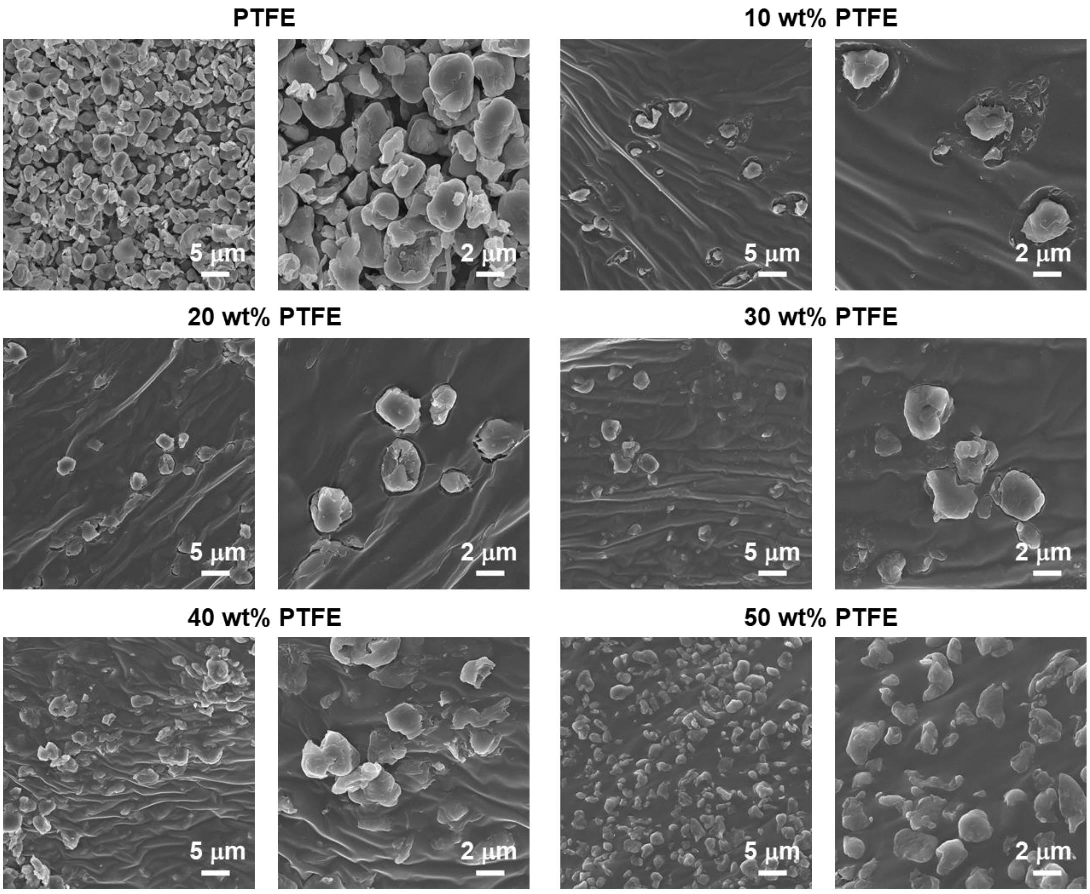

Supplementary Fig. 37. SEM images of the PTFE and PPIL/PTFE.

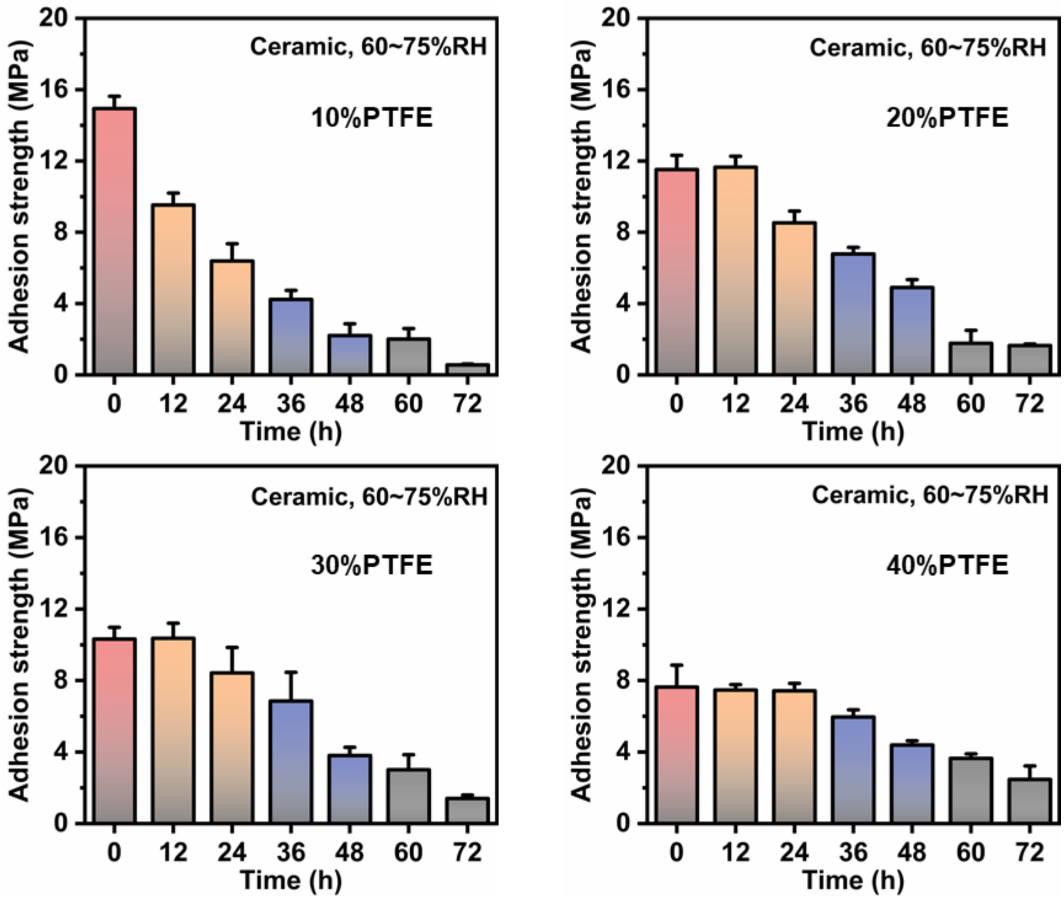

Supplementary Fig. 38. Adhesion strengths of PPIL/PTFE composites after storing in environmental conditions with different humidity. All data are presented as mean ± SD (n = 3~5 independent samples).

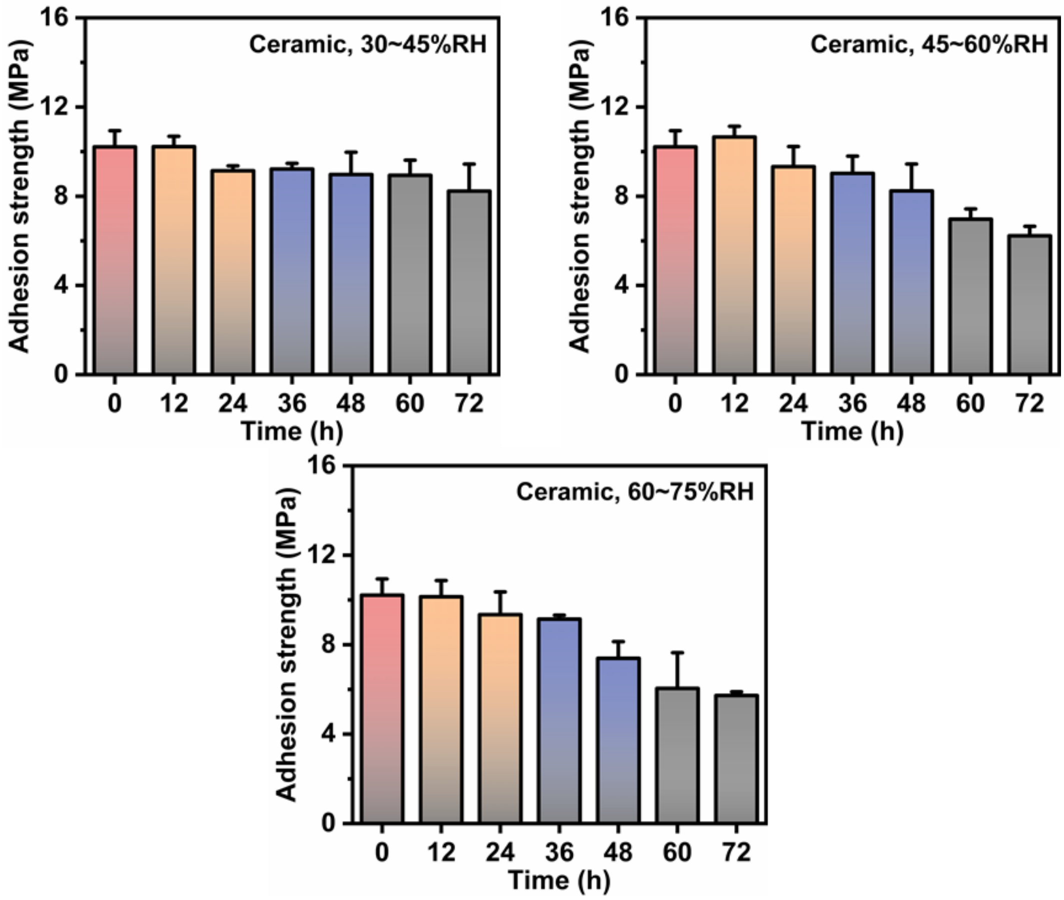

Supplementary Fig. 39. Adhesion strengths of PPIL of P6 after storing in environmental conditions with different humidity. All data are presented as mean ± SD (n = 3~5 independent samples).

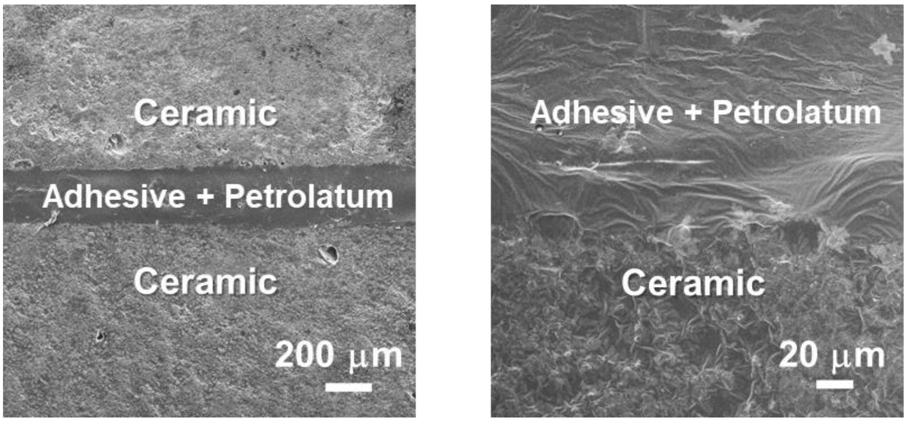

Supplementary Fig. 40. SEM image of the interface between PPIL and the ceramic substrate with petrolatum coating.

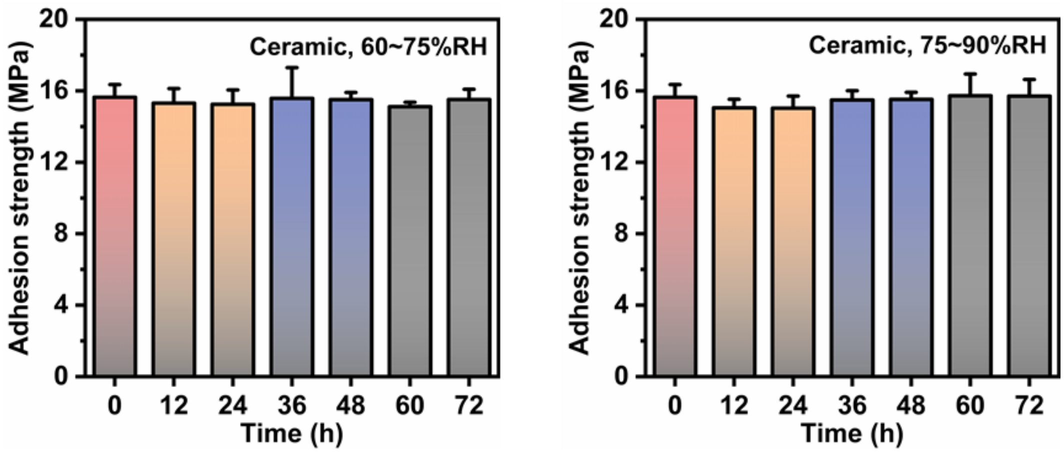

Supplementary Fig. 41. Adhesion strengths of PPIL with petrolatum coating after storing in environmental conditions with different humidity. After storing in a 60 ~ 75%RH environment for 72 h, an adhesion strength of 15.5 MPa was observed (original value: 15.6 MPa). Even if the humidity is further increased to 75 ~ 90%RH, almost no significant reduction in adhesion properties was observed during this process. All data are presented as mean ± SD (n = 3~5 independent samples).

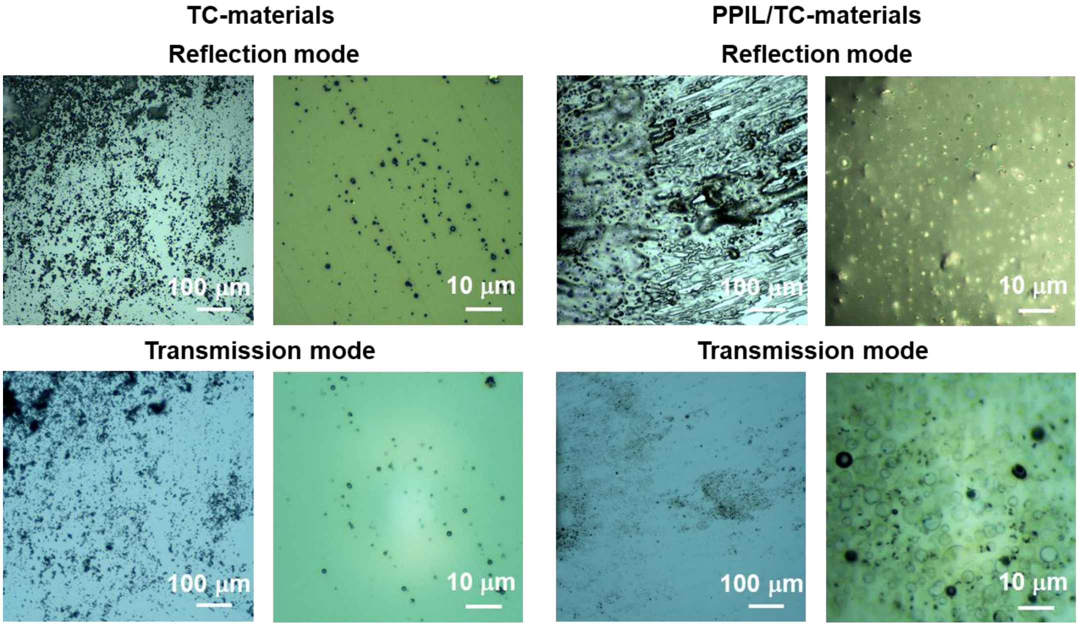

Supplementary Fig. 42. Light microscope (including reflection and transmission mode) images of the thermo-chromic materials and PPIL/thermo-chromic materials.

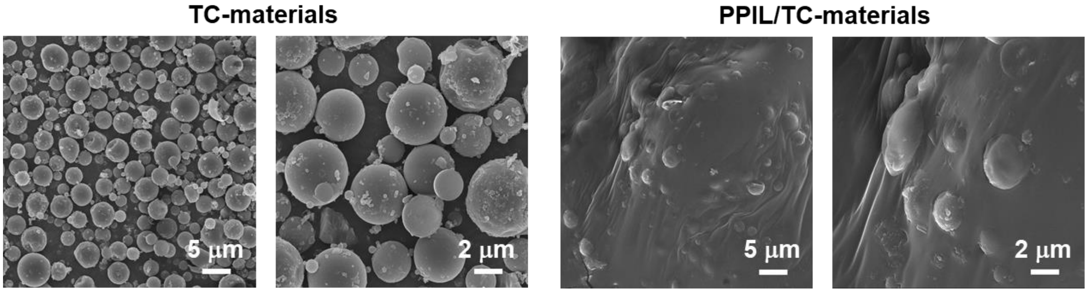

Supplementary Fig. 43. SEM images of the thermo-chromic materials and PPIL/thermo-chromic materials.

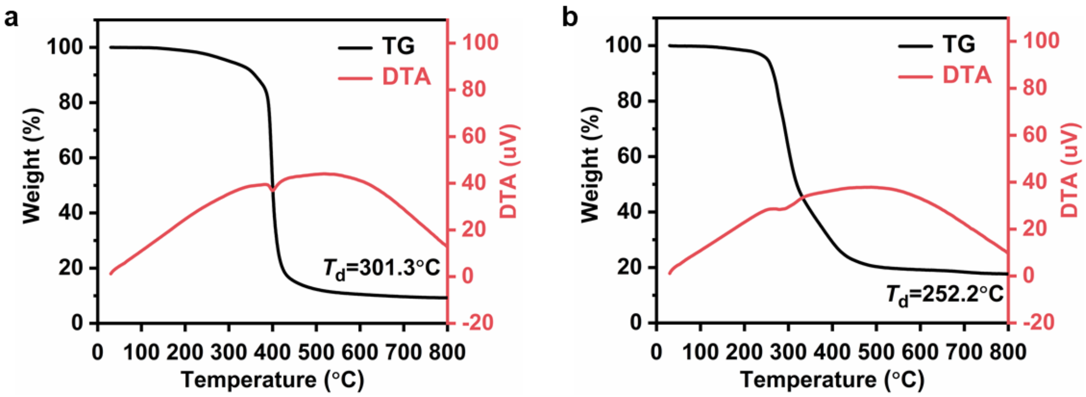

Supplementary Fig. 44. TGA curves. (a) The thermo-chromic materials, (b) PPIL/thermo-chromic materials (T_d is defined as the decomposition temperature when 5% weight loss).

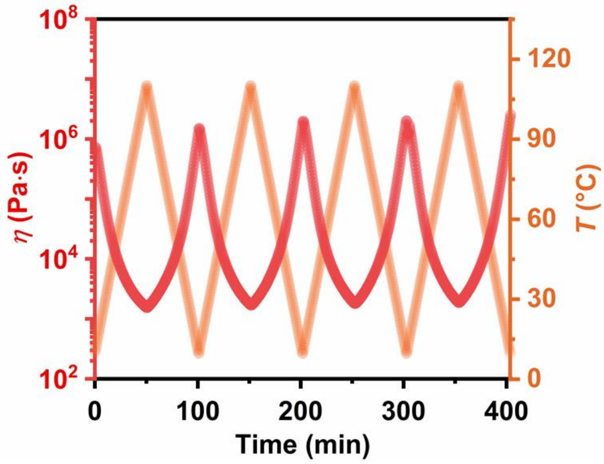

Supplementary Fig. 45. Reversible temperature-dependent rheological test of the PPIL/thermo-chromic materials (angular frequency: 10 rad s^{-1}, strain: 1%).

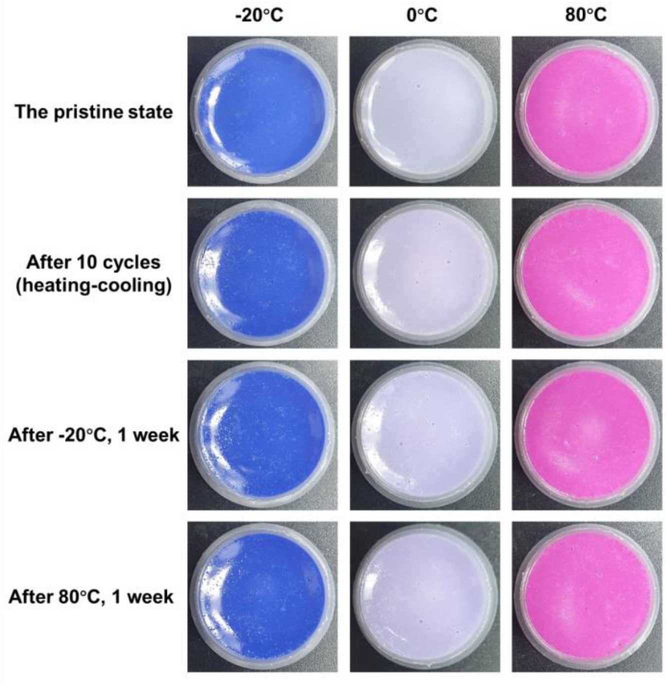

Supplementary Fig. 46. Macroscopic stability test of PPIL/thermo-chromic materials after storing in environmental conditions with different temperatures.

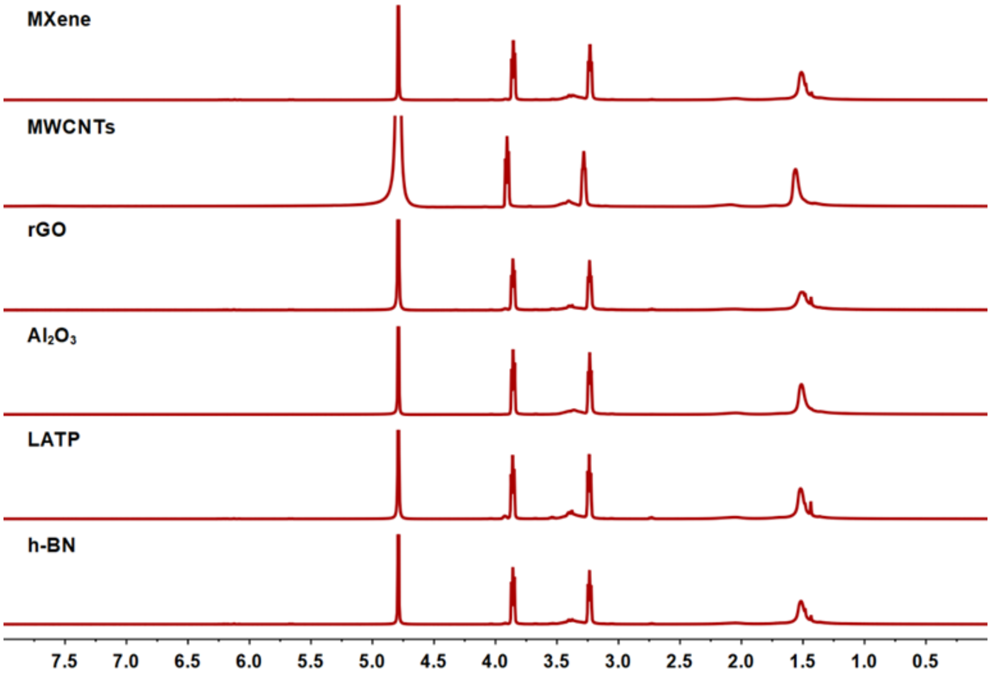

Supplementary Fig. 47. ^{1}H NMR spectrum (400 MHz, 25 ℃, DMSO-d_6) of A2 composite containing various inorganic fillers. No residual NMR signal of vinyl groups indicated that the monomer conversion rates of 100.0% were achieved.

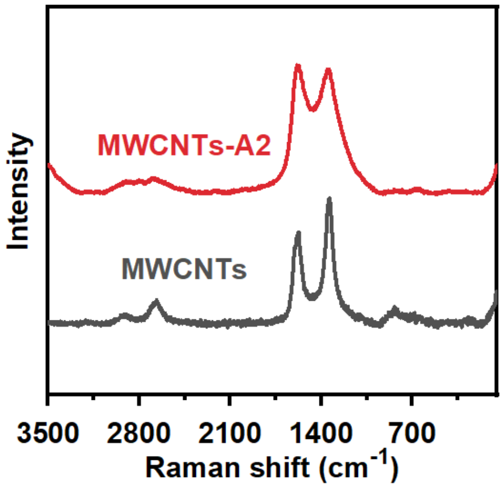

Supplementary Fig. 48. Raman spectra of the MWCNTs and PPIL/MWCNTs.

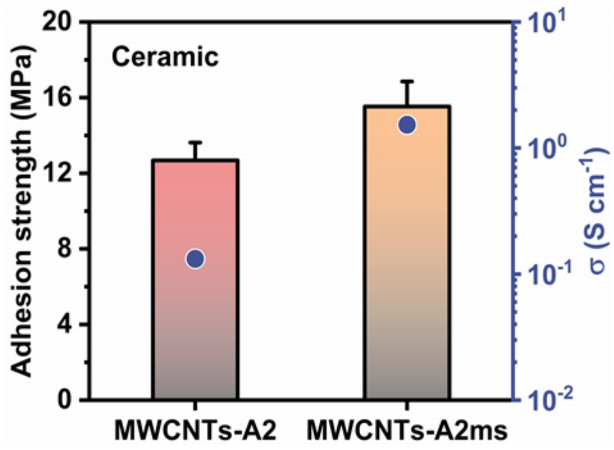

Supplementary Fig. 49. Adhesion strengths and electronic conductivity of A2 composites produced from A2 polymer (MWCNTs-A2) and the in-situ spontaneous polymerization of A2ms (MWCNTs-A2ms). All data are presented as mean ± SD (n = 3~5 independent samples).

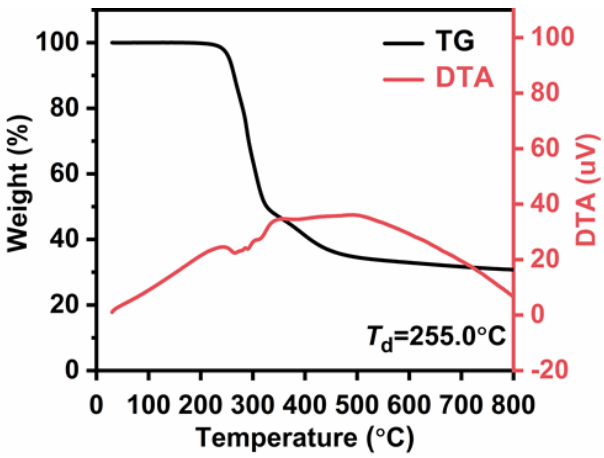

Supplementary Fig. 50. TGA curves of PPIL/MWCNTs (T_d is defined as the decomposition temperature when 5% weight loss).

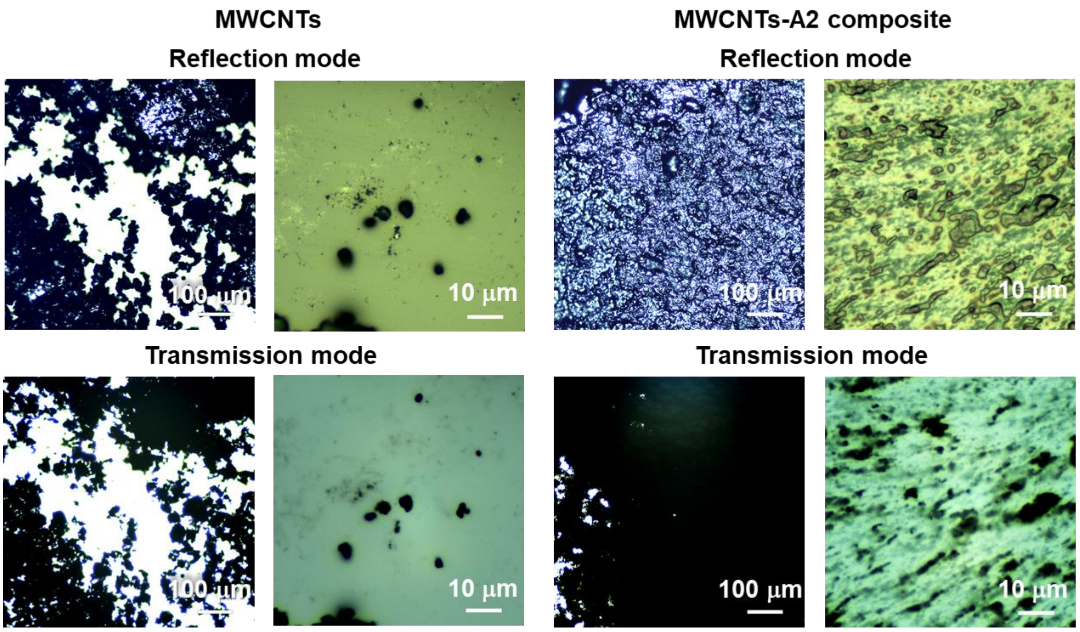

Supplementary Fig. 51. Light microscope (including reflection and transmission mode) images of the MWCNTs and PPIL/MWCNTs.

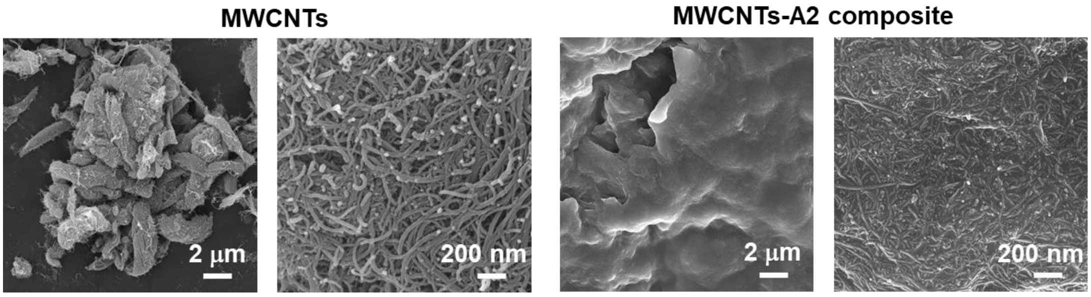

Supplementary Fig. 52. SEM images of the MWCNTs and PPIL/MWCNTs.

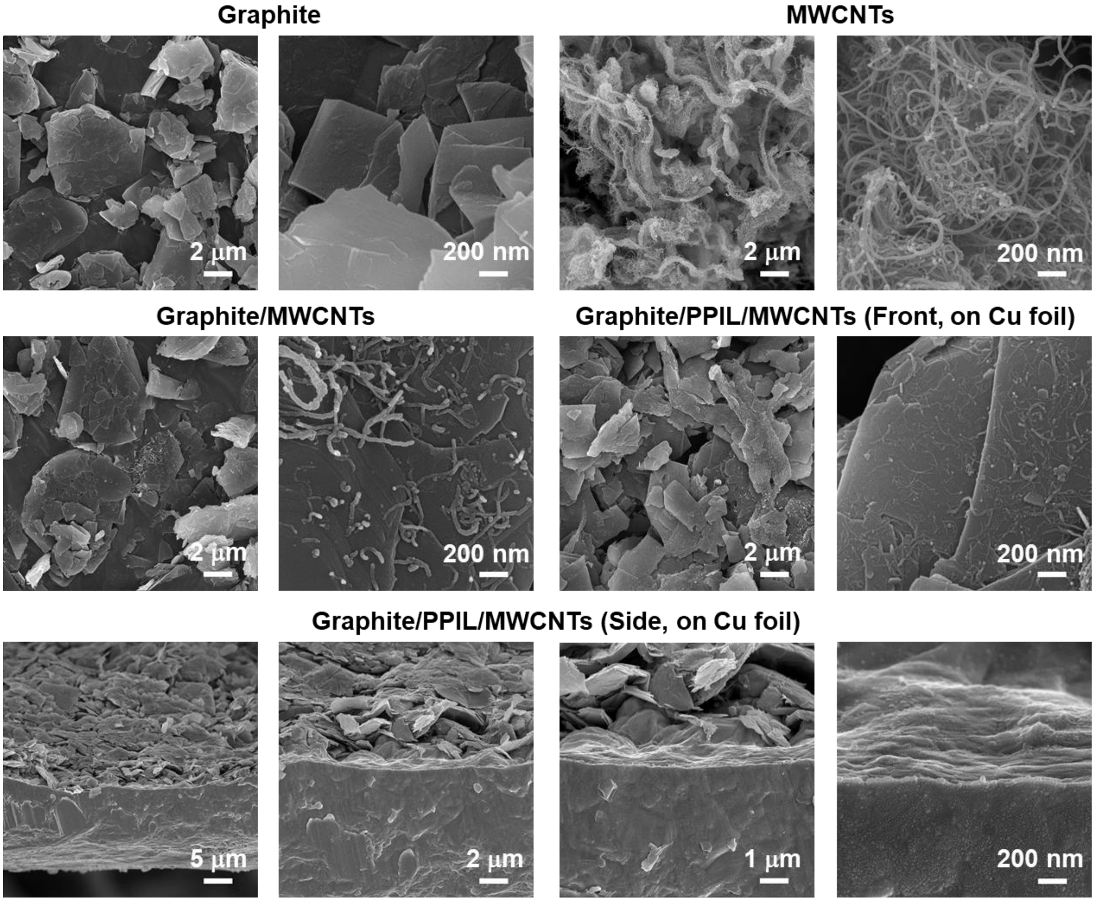

Supplementary Fig. 53. SEM images of the graphite, MWCNTs, graphite/MWCNTs, and graphite/PPIL/MWCNTs on Cu foil (the current collector).

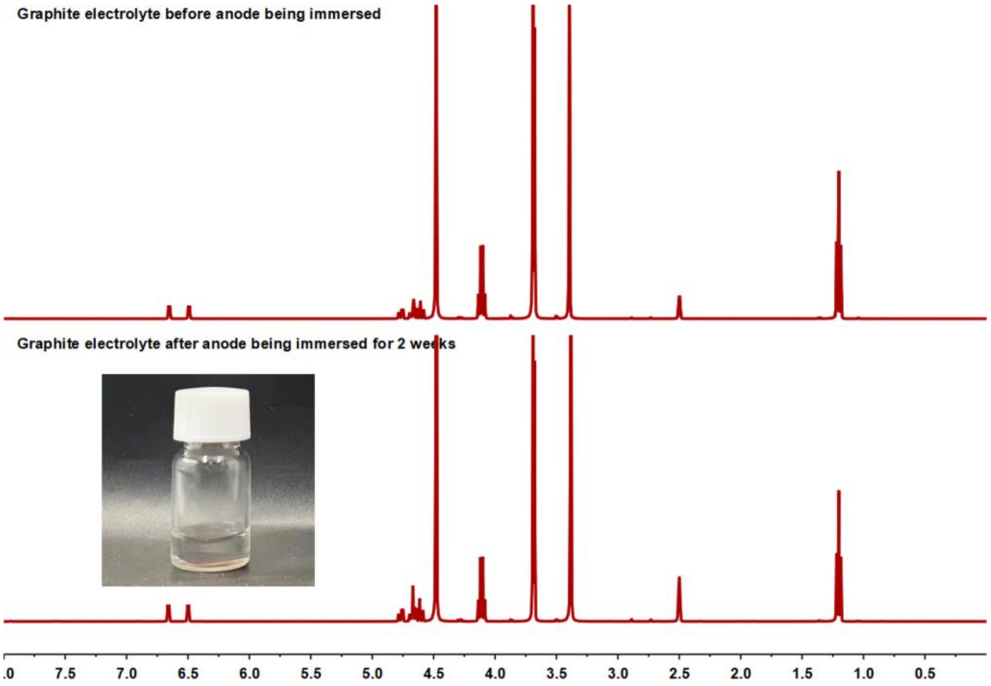

Supplementary Fig. 54. ^{1}H NMR spectrum (400 MHz, 25 ℃, DMSO-d_6) of commercially available graphite electrolytes before and after the anode is immersed for 2 weeks. Immersing the graphite/PPIL/MWCNTs anode in electrolyte for a long time did not result in any network disruption and no NMR signals of the polymeric adhesives were observed.

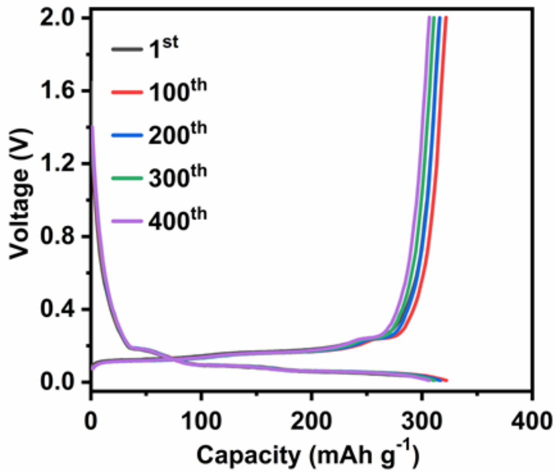

Supplementary Fig. 55. Charge-discharge voltage profiles of the graphite/Li cells at current densities of 2 C.

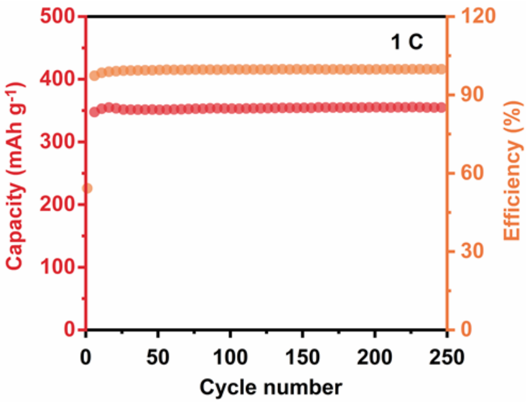

Supplementary Fig. 56. Cycle performance of the graphite/Li cells at current densities of 1 C.

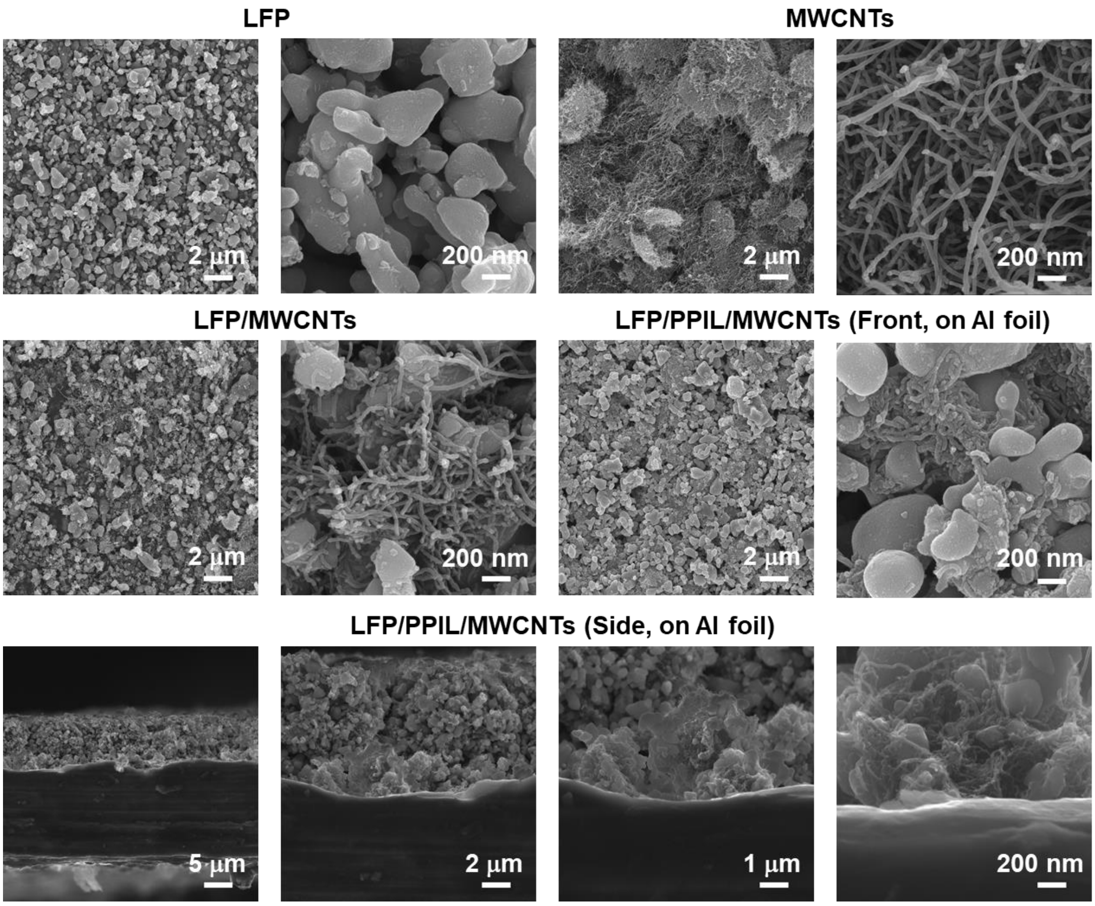

Supplementary Fig. 57. SEM images of the lithium iron phosphate (LFP), MWCNTs, LFP/MWCNTs, and LFP/PPIL/MWCNTs on Al foil (the current collector).

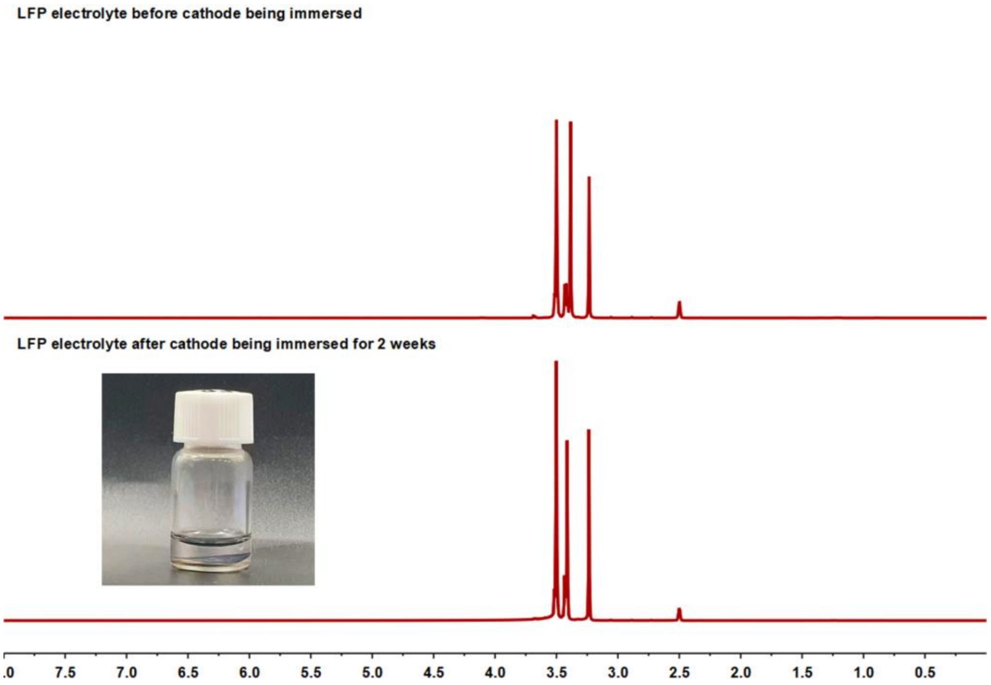

Supplementary Fig. 58. ^{1}H NMR spectrum (400 MHz, 25 ℃, DMSO-d_6) of solvated ionic liquid, i.e., tetraglyme lithium bis(trifluoromethanesulfonyl)amide, electrolytes before and after the cathode is immersed for 2 weeks. Immersing the LFP/PPIL/MWCNTs cathode in electrolytes for a long time did not result in any network disruption and no NMR signals of the polymeric adhesives were observed.

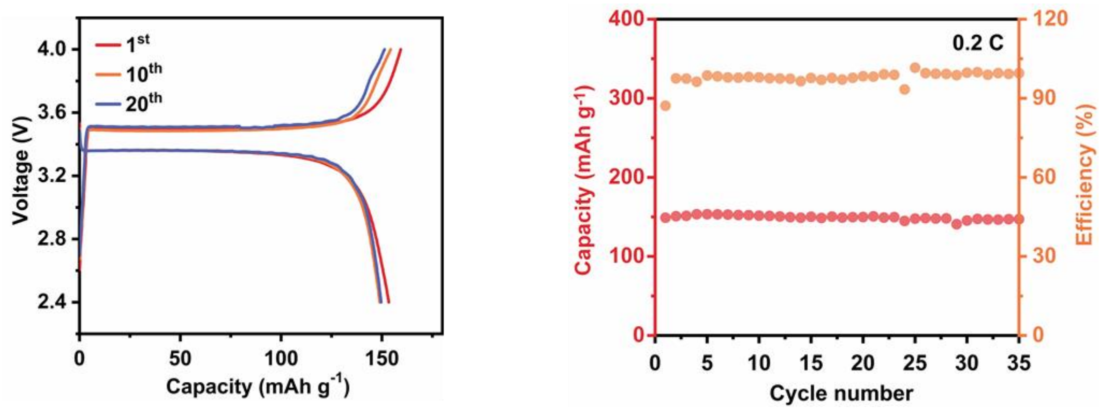

Supplementary Fig. 59. Charge-discharge voltage profiles and cycle performance of the LFP/Li cells at current densities of 0.2 C.

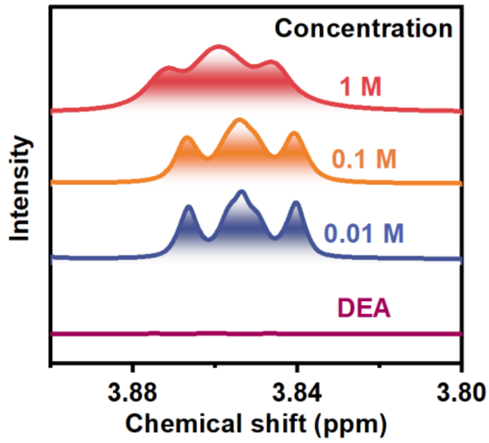

Supplementary Fig. 60. Concentrations-dependent ^{1}H NMR spectra of solutions of spontaneously polymerized A2 (solvent: D_2O).

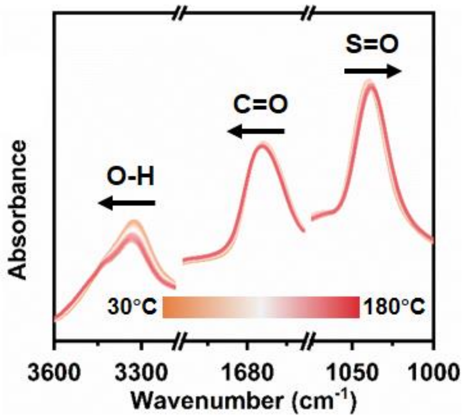

Supplementary Fig. 61. Temperature-dependent FT-IR spectra of A2 upon heating from 30 to 180 ℃ (interval: 10 ℃).

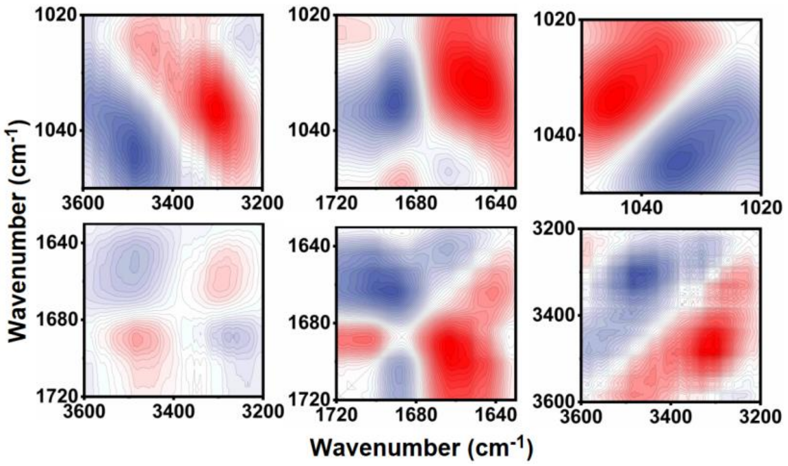

Supplementary Fig. 62. Two-dimensional COS asynchronous spectra of A2 generated from temperature-dependent FT-IR spectra. In the contour maps, red and blue colors are defined as positive and negative intensity, respectively.

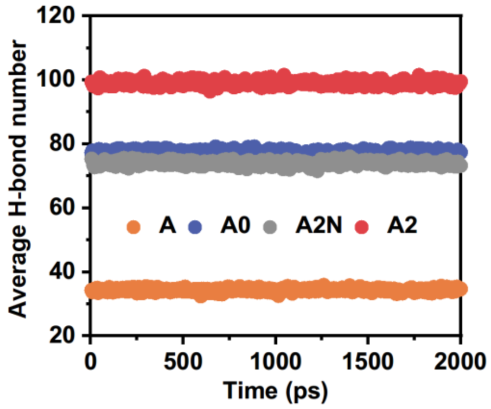

Supplementary Fig. 63. Average H-bond number of A2 and reference samples.

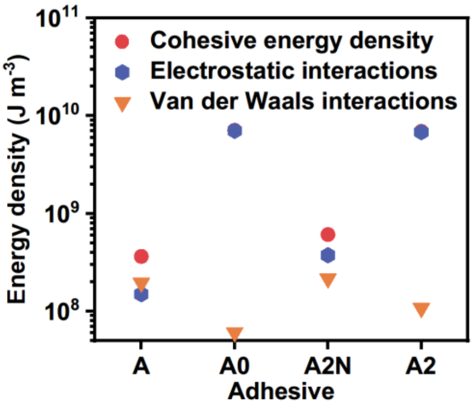

Supplementary Fig. 64. The composition of theoretically calculated CED. Van der Waals force is the reflection of the attractive and the repulsive non-bond forces between molecules. Electrostatic interactions describe the attraction between polar or completely ionized groups, including ionic interactions, and H-bonding interactions. Electrostatic interactions of PPILs (e.g., A0 and A2) were much higher than that of nonionic analogs (e.g., A and A2N).

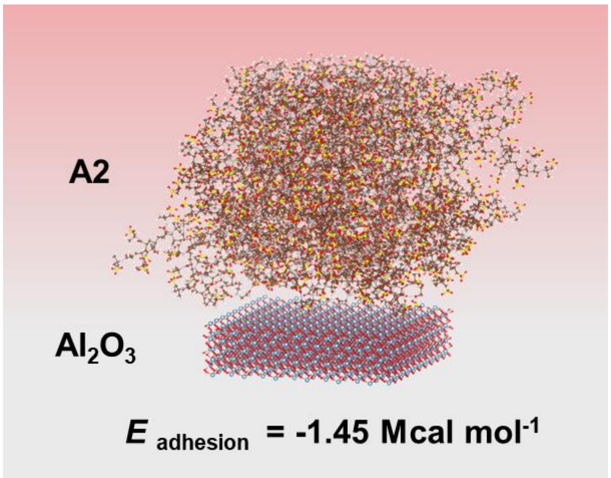

Supplementary Fig. 65. The most stable conformation with a minimum energy of A2 on ceramic (Al_2O_3) substrate.

Supplementary Table 1. Molecular weights (M_w) and polydispersity indices (PDI, calculated from M_w/M_n) of the spontaneously polymerized PPILs and reference samples with partial disentangled (DE-A2) or polymerized using initiator of ammonium persulfate (APS-A2).

	A	A0	A1	A2	A3	A2N	DE-A2	APS-A2
M_W	217090	144264	125291	106987	113983	46134	133155	68759
PDI	4.192	9.749	4.478	3.186	6.299	4.661	5.752	1.698

Supplementary Table 2. Comparison of adhesion strength of PPILs and other reported functional adhesives at low temperatures.

Adhesive	Temperature (°C)	Adhesion strength (MPa)	Reference
PC10-W1	-196	1.2	*J. Am. Chem. Soc.* 2020, 142, 21522
TA-epoxy	-196	0.6	*Biomacromolecules* 2022, 23, 3493
CA/PEG2000	-196	0.9	*ACS Appl. Polym. Mater.* 2022, 4, 4319
Poly(PA-H)	-60	0.4	*ACS Appl. Mater. Interfaces* 2022, 14, 27476
C12TAB/ChCl-urea	-80	1.0	*Mater. Horiz.* 2022, 9, 1700
BSA0.35	-196	9.5	*Adv. Sci.* 2022, 220318
P1	-196	4.8	*ACS Nano* 2022, 16, 5303
NIPA5.0	-196	15.9	*Chem. Mater.* 2023, 35, 7730
6-HTPB	-80	2.1	*Green Chem.* 2023, 25, 6845
PEA	-196	5.6	*Eur. Polym. J.* 2023, 198, 112387

Poly(A-C)	-196	9.5	*Chem. Eng. J.* 2023, 451, 138674
Poly(TA-DB)	-65	7.9	*Chem. Eng. Sci.* 2023, 281, 119164
cPA2	-196	17.4	*J. Mater. Chem. A* 2023, 11, 6286
DPETI	-196	2.2	*Adv. Mater.* 2023, 2310779
A2	-196	10.0	This work

Supplementary Table 3. Comparison of organic solvent resistance of PPILs and other reported functional adhesives.

Adhesive	Types of organic solvents	Soaking time	Adhesion strength after soaking (MPa)	Reference
VPTA	2	22 d	N/A	*J. Mater. Chem. A* 2017, 5, 21169
DPU-HMA	1	18 h	2.7	*Mater. Chem. Front.* 2019, 3, 1833
CT-2	5	21 d	2.7	*CCS Chem.* 2020, 2, 1690
SP-DN	7	24 h	0.8	*Mater. Horiz.* 2021, 8, 2520
P4-AS-PAA	2	6 m	0.9	*Adv. Funct. Mater.* 2021, 2109144
HPU-HMA	3	30 d	N/A	*Ind. Eng. Chem. Res.* 2021, 60, 6925
P1	9	100 d	4.8	*ACS Nano* 2022, 16, 5303
C12TAB/ChCl-urea	4	N/A	0.6	*Mater. Horiz.* 2022, 9, 1700

BSA0.35	3	24 h	14.6	*Adv. Sci.* 2022, 9, 2203182
PVA-PTA	2	30 min	N/A	*Adv. Funct. Mater.* 2022, 2111892
PEG-TA	2	7 d	4.0	*Macromol. Rapid Commun.* 2022, 43, 2100830
NIPA5.0	9	168 h	18.8	*Chem. Mater.* 2023, 35, 7730
6-HTPB	4	4 h	1.1	*Green Chem.* 2023, 25, 6845
A2	8	1 m	12.8	This work

Supplementary Table 4. Comparison of adhesion strength and curing temperature of PPILs and other reported functional adhesives.

Adhesive	Curing temperature (°C)	Adhesion strength (MPa)	Reference
P(VA-g-HBA)	140	17.3	*Macromol. Rapid Commun.* 2016, 37, 545
LC	100	1.6	*Nat. Commun.* 2016, 7, 12094
PDMS-COO-Zn	70	6.8	*Nat. Commun.* 2018, 9, 2725
BSA	95	4.0	*J. Am. Chem. Soc.* 2019, 141, 1359
DPU-HMA	130	5.7	*Mater. Chem. Front.* 2019, 3, 1833
2e+MX-154	205	20.5	*Angew. Chem. Int. Ed.* 2019, 58, 12271
SP-H-SB1%	120	2.0	*Green Chem.* 2020, 22, 1319
SEA0.2	80	10.2	*ACS Materials Lett.* 2021, 3, 7, 1003

IC-1	100	5.8	*Angew. Chem. Int. Ed.* 2021, 60, 8948
SiNP S-Bpin	215	39.6	*Sci. Adv.* 2021, 7, eabk2451
PTBN6	90	4.2	*Adv. Funct. Mater.* 2022, 2201959
BSA0.35	100	14.6	*Adv. Sci.* 2022, 9, 2203182
PGA-3	120	7.0	*Adv. Mater.* 2023, 35, 2300802
Soy-mal-tan	180	15.0	*Nature* 2023, 621, 306
A2	110	16.2	This work

Supplementary Table 5. Comparison of adhesion strength and adhesion area of PPILs and other reported functional adhesives.

Adhesive	Substrate	Area (mm * mm)	Adhesion strength (MPa)	Reference
2e+MX-154	Al	10 * 10	20.5	*Angew. Chem. Int. Ed.* 2019, 58, 12271
$(A)_n$	Glass	2.5 * 2.5	4.2	*J. Am. Chem. Soc.* 2020, 142, 2579
IC-1	Glass	5 * 5	5.8	*Angew. Chem. Int. Ed.* 2021, 60, 8948
pIG2-N0.4	Glass	15 * 10	4.7	*Mater. Horiz.* 2021, 8, 2057
SiNP S-Bpin	Glass	3 * 3	39.6	*Sci. Adv.* 2021, 7, eabk2451
P4-AS-PSS	Ceramic	20 * 15	1.2	*Adv. Funct. Mater.* 2021, 2109144
BSA0.35	SS	25 * 12.5	14.6	*Adv. Sci.* 2022, 9, 2203182

PVA/BA	Glass	10 * 10	0.6	*PNAS* 2022, 119, e2203074119
BN-6	SS	10 * 4	4.2	*Adv. Funct. Mater.* 2022, 2201959
ADM-4CTAB	SS	10 * 20	3.1	*Angew. Chem. Int. Ed.* 2022, e202204611
3I	SS	10 * 10	0.5	*Adv. Mater.* 2023, 35, 2208413
BSA	Titanium	10 * 10	4.0	*Nat. Commun.* 2023, 14, 5145
TFMD-2	Glass	10 * 10	1.2	*Adv. Funct. Mater.* 2023, 2304653
Soy-mal-tan	Al	12 * 12	15.0	*Nature* 2023, 621, 306
A2	Ceramic	10 * 10	16.2	This work

Supplementary Table 6. Solvents-induced swelling tendency of A2 when it was immersed in the organic solvents for 1 month.

Solvent	n-Hexane	DCM	EAC	THF	EtOH	Acetone	IPA	ACN
Swelling tendency (%)	0.39	0.10	0.19	0.05	2.23	0.33	0.10	0.29

Swelling tendency was calculated using the following equation: swelling tendency = $(M_s - M_d) / M_d \times 100\%$, wherein M_s is the wet mass of swelling and M_d is the dry mass of the initial. Each test was carried out 3 times and averaged.

Supplementary Table 7. Results of the multiplication of the signs of each cross-peak in 2DCOS synchronous and asynchronous spectra of A2.

1028	–	–	–	–	–	
1045	+	+	+	–		
1646	+	+	+			
1689	+	+				
3301	+					
3525						
	3525	3301	1689	1646	1045	1028

According to Noda's rule, the final specific order for A2 during heating is given as follows: 1028 (disassociated S=O) → 3525 (disassociated O-H) → 3301 (associated O-H) → 1689 (disassociated C=O) → 1045 (associated S=O) → 1646 cm^{-1} (associated C=O).
