# Supplementary material for: Concentration-induced spontaneous polymerization of protic ionic liquids for efficient in situ adhesion
Source: Nat Commun. 2024 May 20;15:4265. doi: 10.1038/s41467-024-48561-1 (PMC11106314; doi:10.1038/s41467-024-48561-1)
Supplement: Supplementary file 3 — Description of Additional Supplementary Files [file 41467_2024_48561_MOESM3_ESM.pdf]

Description of Additional Supplementary Files

File Name: Supplementary Data 1
Description: Molecular dynamics trajectories of A, AWN, AO, A2, and A2-ceramic.

File Name: Supplementary Movie 1
Description: Flammability test.

File Name: Supplementary Movie 2
Description: Macroscopic adhesion test of A2 on ceramic substrate.
